# Exposure of insects to current use pesticide residues in soil and vegetation along spatial and temporal distribution in agricultural sites

**DOI:** 10.1038/s41598-024-84811-4

**Published:** 2025-01-21

**Authors:** Carolina Honert, Ken Mauser, Ursel Jäger, Carsten A. Brühl

**Affiliations:** iES Landau, Institute for Environmental Sciences, University of Kaiserslautern-Landau, Landau, Germany

**Keywords:** Synthetic pesticide, Non-target organism, Mixture, Chronic, Environmental chemistry, Environmental impact, Biodiversity, Agroecology

## Abstract

Current use pesticides (CUPs) are recognised as the largest deliberate input of bioactive substances into terrestrial ecosystems and one of the main factors responsible for the current decline in insects in agricultural areas. To quantify seasonal insect exposure in the landscape at a regional scale (Rhineland-Palatine in Germany), we analysed the presence of multiple (93) active ingredients in CUPs across three different agricultural cultivation types (with each three fields: arable, vegetable, viticulture) and neighbouring meadows. We collected monthly soil and vegetation samples over a year. A total of 71 CUP residues in different mixtures was detected, with up to 28 CUPs in soil and 25 in vegetation in single samples. The concentrations and numbers of CUPs in vegetation fluctuated over the sampling period, peaking in the summer months in the vegetation but remaining almost constant in topsoil. We calculated in-field additive risks for earthworms, collembola, and soil-living wild bees using the measured soil concentrations of CUPs. Our results call for the need to assess CUP mixture risks at low concentrations, as multiple residues are chronically present in agricultural areas. Since this risk is not addressed in regulation, we emphasise the urgent need to implement global pesticide reduction targets.

**Supplementary Information:**

The online version contains supplementary material available at 10.1038/s41598-024-84811-4.

## Introduction

Pesticides are chemicals used to prevent, destroy, repel, or mitigate pests. They can also be used as plant regulators, defoliants, or desiccants^1^. The application of pesticides is recognised as the largest intentional input of biologically active substances into terrestrial ecosystems^2^. Synthetic current use pesticides (CUPs) have been detected not only in cultivated land^2–5^ but also in nontarget areas^6–12^ and at the landscape level^13–15^.

In recent decades, a decline in aerial insect biomass was observed in both, agricultural landscapes^16^ and nature reserves^17^ in Germany. Many factors, such as climate change, landscape fragmentation and agricultural intensification, including the use of pesticides, are considered stressors of the observed decline^18,19^. However, in a pan-European study that measured the in-field biodiversity of plants, insects and birds, pesticides were identified as stressors with greater explanatory value than structural landscape features or fertiliser use^20^.

On cultivated land, a series of CUPs are applied throughout the year. This results in the accumulation of multiple substances. This realistic mixture exposure is not consistently assessed in environmental risk assessment procedures for regulation, as a comprehensive mixture risk assessment is generally not performed. While individual tank mixtures, if listed on the label of the product with a clear name and dose rate and formulations are tested^21^, the actual realistic mixtures are not considered^22^. In addition, the current insect exposure assessment does not adress soil or vegetation as sources of contamination^23^, despite its acknowledged relevance^24^. Toxicological effect data on pesticide exposure in soil are available for earthworms and collembolans. Recent research has focused on evaluating the potential risks associated with pesticides for pollinators, regarding the presence of CUPs in pollen and nectar and a revised EFSA document was published^25^. Besides the fact, that bumble bees and many solitary bees build their nests in soil^26^, exposure in the risk assessment by pesticides in soil is only considered through pollen and nectar contaminations via soil^25,27,28^. Moreover, there is a lack of data on the toxicological endpoints resulting from pesticides on above-ground plant material, like stems and leaves, despite various insects using them as nesting material, living on them, or consuming them as larvae (e.g. caterpillars) or adult (e.g. grasshoppers).

Various pesticide mixtures have been measured in recent years in agricultural soils^2–5,8,11,29,30^ and non-agricultural soils^5,31^ as well as in the vegetation of nontarget areas^7,9,10,32^ using multicomponent analytical methods. During a study in Switzerland, agricultural soil samples with 14 years of pesticide history were investigated^33^. Their results indicate that the presence of CUPs goes beyond recent applications, forming a substantial background of various residues in the soil. However, as in most studies, the samples were collected once a year. Thus, it remains unclear how CUP residues change throughout the season, affecting the exposure of non-target organisms.

To investigate the realistic exposure of insects to CUPs in landscapes characterised by agriculture, we sampled and analysed two environmental matrices, soil and vegetation. Samples were taken from nine different agricultural sites (arable management system, vegetable management system and viticulture, each *n* = 3) separated by 0.20 to 41.8 km in Rhineland-Palatine, Germany, with an adjacent meadow to each site (for further site details see Supplementary Table S16 and S16b). We sampled every month for an entire year to be able to link exposure to the activity patterns of different insects.

The objectives of this study were (1) to quantify the seasonal variation in synthetic CUP mixtures in soil and vegetation by monthly sampling over one year; (2) to characterise spatial in-field and off-field (adjacent meadows) CUP distributions at different distances (1, 5, and 20 m from the field to the meadow); (3) to calculate the risk of realistic CUP mixture exposure for earthworms, collembolans and a surrogate ground-nesting wild bee. Our research hypotheses were that (a) CUPs fluctuate seasonally in soil and vegetation in numbers and concentration and (b) decrease with distance from the agricultural fields, with (c) far-reaching transportation in 3D (viticulture) compared to 2D (arable and vegetable) applications.

## Results

### CUP mixtures in soil and vegetation

Of the 93 CUPs analysed, 66 (71%, 30 fungicides (F), 23 herbicides (H) and 13 insecticides (I)) were detected in the topsoil samples (see Supplementary Table S1). Each analysed topsoil sample (*n* = 468) contained at least one CUP, 94% contained two or more CUPs and in 53% of all topsoil samples, ten or more CUPs were recorded. On average ten CUPs were recorded in soil samples. A maximum of 28 different CUPs was detected in a single topsoil sample from an arable (winter wheat) field in October (see Supplementary Table S2). The most frequently occurring substances per pesticide group in topsoil samples were the fungicide (F) fluopyram, the herbicides (H) terbuthylazine and pendimethalin) and the insecticide (I) chlorantraniliprole (Table 1). The topsoil samples revealed 349 different CUP mixture combinations with 124 mixtures containing at least one insecticide. The most common mixture of azoxystrobin (F) and fluopyram (F) in all the soil samples occurred 11 times. In the soil, the mean concentration of 29 of the 66 recorded CUPs was < 1 µg/kg. The highest mean concentration for single CUPs in topsoils was detected for metrafenone (F, 26.53 µg/kg see Supplementary Table S1). However, some CUPs, e.g. s-metolachlor (H), azoxystrobin (F) and pendimethalin (H), were detected at concentrations > 500 µg/kg (see Supplementary Table S1). The maximal concentration was recorded for pendimethalin (H, 981.18 µg/kg, see Supplementary Table S1, vegetable field (celery), in-field (−20 m), May 2021)^34^).

Sixty-two of the 93 CUPs (67%, 31 fungicides, 20 herbicides and 11 insecticides; Supplementary Table 1) were detected in the vegetation samples. Almost all vegetation samples (97%, *n* = 392) contained at least one CUP, with 89% containing two or more CUPs. The average number of CUPs recorded in the vegetation samples was seven (see Supplementary Table S2). In 30% of the samples, ten or more CUPs were recorded. Up to 25 different CUPs were measured in two vegetation samples (both celery cultivation) from vegetable fields (June and July 2021). Fluopyram (F) and chlorantraniliprole (I) are the most commonly detected fungicides and insecticides (Table 1). Prosulfocarb was the most prevalent herbicide recorded in vegetation (Table 1). In the vegetation samples, 344 CUP combinations were recorded, with 68 mixtures containing at least one insecticide. The most frequent mixture was azoxystrobin (F) and fluopyram (F), which occurred in seven samples. In the vegetation, the mean concentration of 7 CUPs of 68 was < 1 µg/kg and between 1 and 10 µg/kg for 27 CUPs. The highest mean concentration of single CUP in vegetation was recorded for fludioxonil (F, 1,349.55 µg/kg see Supplementary Table S1). The maximal concentration of 67,656.10 µg/kg was measured for dimethomorph (F, viticulture, in-field (−20 m), July 2021).

**Table 1:** Detection frequency, total mean concentration [µg/kg] and maximum concentration [µg/kg] of the three most commonly detected CUPs in soil and vegetation (in bold).

	Detection frequency (%)		Total mean conc. [µg/kg]		Maximum conc. [µg/kg]	
	Soil (*n* = 468)	Vegetation (*n* = 442)	Soil (*n* = 468)	Vegetation (*n* = 442)	Soil (*n* = 468)	Vegetation (*n* = 442)
Fungicides						
Fluopyram	**93.6**	**70.4**	6.04	28.79	164.62	954.42
Herbicides						
Pendimethalin	**28.2**	16.1	24.22	463.34	981.18	23,161.44
Prosulfocarb	14.1	**35.1**	0.27	4.05	0.78	28.15
Terbuthylazine	**28.2**	12.2	2.34	11.80	205.10	218.52
Insecticides						
Chlorantraniliprole	**14.3**	**10.2**	3.15	102.73	27.09	3,988.48

**Fig. 1:**
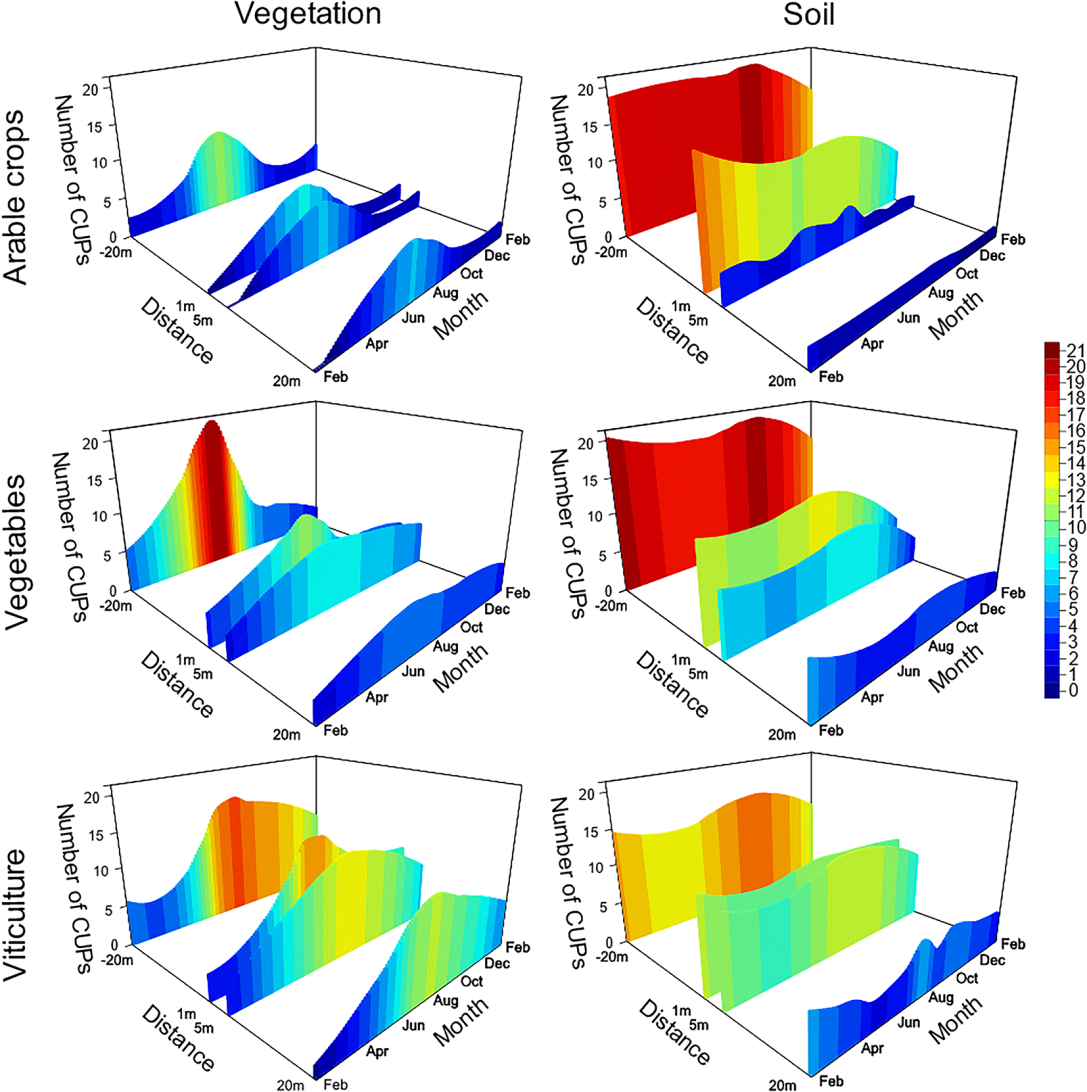
Number of CUPs throughout the year in soil (left graphs) and vegetation (right graphs) from different agricultural sites (arable crops, vegetables, and viticulture) at four different measurement points (in the target area (−20 m) and off-field in an adjacent meadow (1 m, 5 m and 20 m from the field margin)) over 13 months from February 2021 to February 2022. The colours indicate the number of CUPs. The data were modelled based on the raw data with a loess smooth.

### Temporal CUP distribution

#### Temporal distribution of CUPs in soil of different crops

The number of CUP residues in topsoils remained nearly constant throughout the year. In arable fields, the CUP numbers were constant until October, when a maximum was detected, afterwards the number of CUPs slightly decreased. In-field at vegetable sites, two maxima were detected (February and October 2021, Fig. 1, see Supplementary Table S3), with a minimum in May. The same was calculated in-field at viticulture sites (Fig. 1, see Supplementary Table S3). The temporal course of the CUP numbers in topsoil samples from target areas (fields) differed only slightly among the three cultures (KL divergence ≤ 0.01, see Supplementary Table S4).

#### Temporal distribution of CUPs in the vegetation of different crops

Seasonal fluctuations in CUP numbers were pronounced in the vegetation (Fig. 1). In the field, the number of CUPs increased until the middle of summer. Maxima in-field from vegetation samples were detected for all managment systems in July and August (see Supplementary Table S3) and again in October for vegetables. Concerning arable and vegetable crops, similar trends were observed (KL-divergence see Supplementary Table S5) the number of CUPs decreased after the peak and remained high in viticulture (Fig. 1). In the off-field, the number of CUPs remained higher at the viticulture sites compared to the other cultivation types (Fig. 1).

### Spatial CUP distribution

**Fig. 2:**
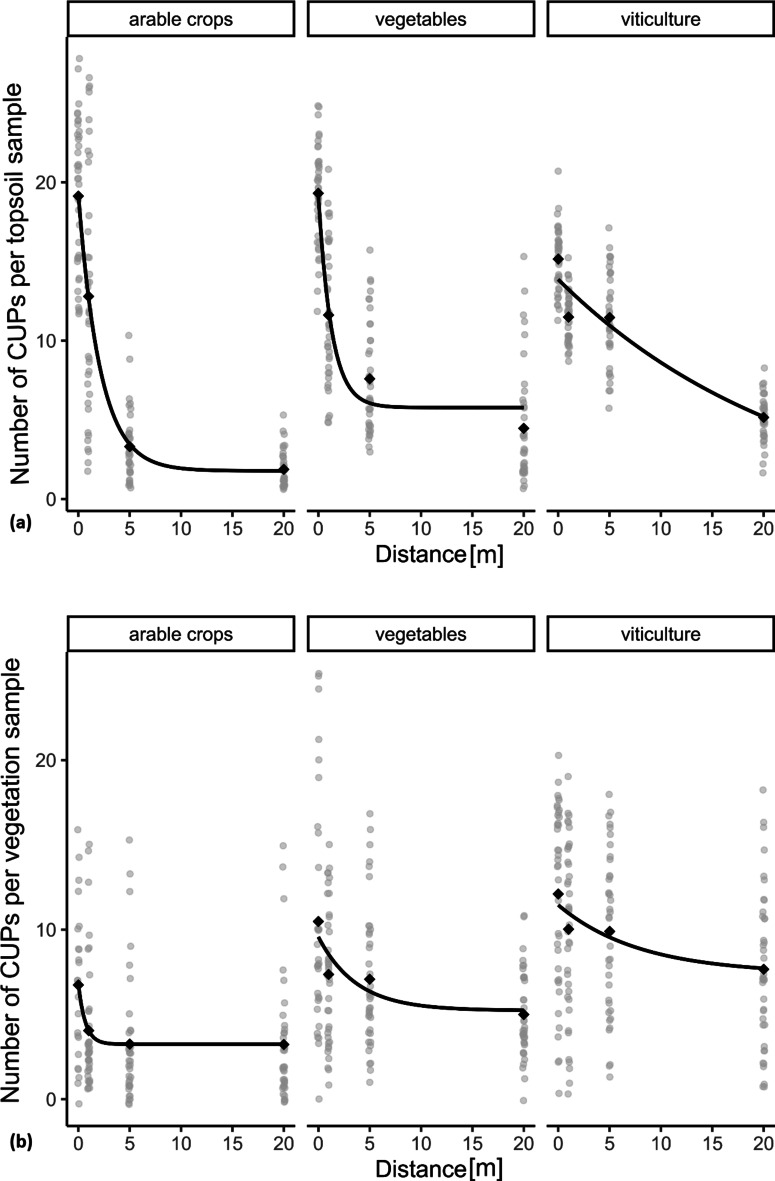
Number of CUP per sample with increasing distance to the field for all investigated management systems (arable crops, vegetables and viticulture) in soil (**a**) and vegetation (**b**). CUP number per sample and distance. The curves show the exponential fit with the R-function nlsLM^35^. All samples (vegetation: *n* = 442, soil: *n* = 468) collected throughout the year are included. The in-field samples were labelled as distance 0 and the off-field samples were collected at distances of 1, 5 and 20 m in an adjacent meadow.

An exponential function was used to fit the data and analyse the drift for the different cultivation types. A consistent decrease in CUPs was observed from in-field to meadow for all land use types in terms of soil and vegetation (Fig. 2).

#### CUPs in soil of different management systems

##### Arable crops

In topsoil from arable fields, an average of 19 CUPs was detected (see Supplementary Table S6). A decrease in the number of CUPs (1 m: 13 CUPs, 5 m: 3 CUPs, 20 m: 2 CUPs, see Supplementary Table S6) was measured at the arable sites with increasing distance (b= −0.47, see Supplementary Table S7, Fig. 2). Insecticides were only recorded in the field and 1 m from the field edge, represented by 6 and 4 substances, respectively (see Supplementary S8).

##### Vegetables

In the field, at the vegetable sites (−20 m), an average of 19 CUPs was recorded (see Supplementary Table S6). A decrease in CUPs with increasing distance was measured off-field (Fig. 2, see Supplementary Table S7: b= −0.52 and see Supplementary Table S6: 1 m: 12 CUPs, 5 m: 8 CUPs, 20 m: 5 CUPs). Over all months, eleven different insecticides were detected in the soils from the vegetable fields. At 1 m off-field, six insecticides were detected, three at 5 m and one at 20 m (see Supplementary Table S8).

##### Viticulture

On average, 15 CUPs were detected in the in-field topsoil at the viticulture sites, decreasing to 12 CUPs at 1 m as well as 5 m and 5 CUPs at 20 m (see Supplementary Table S6). In viticulture, a greater drift into the off-field area was measured compared to arable and vegetable crops (Fig. 2, see Supplementary Table S7: b = − 0.11). The 20 m measurement point at one of the three viticulture sites was situated in a nature conservation area. During the main spraying season, a maximum of 7 CUPs was recorded at this point in August (May: 2 CUPs, June: 2 CUPs, July 4 CUPs). Overall, viticulture sites were dominated by fungicides: residues of 23 different fungicides, and two herbicides and two insecticides were detected in topsoil samples inside the vineyards (see Supplementary Table S8 and Figure S1).

#### CUPs in vegetation of different management systems

In 14 vegetation samples, no CUP was detected (seven samples at 20 m, six at 5 m and one at 1 m off-field). Twelve of the 14 samples without CUP detection were collected at arable sites (at 5 and 20 m), one at the vegetable site (20 m, February 2022) and one at the viticulture site (1 m, February 2021).

##### Arable crops

On average, 7 CUPs were detected in vegetation in arable crops (see Supplementary Table S6). A decrease in CUP numbers from the cultivated area to the meadow (Fig. 2, see Supplementary Table S7: b = − 1.45) was measured. On average, 4, 4 and 3 CUPs were detected in meadow vegetation samples at 1 m, 5 m and 20 m off-field, respectively (see Supplementary Table S6). Fungicides were detected most predominantly, followed by herbicides and insecticides. Three different insecticides (acetamiprid, thiamethoxam at 5 m, spinosad and thiamethoxam at 20 m; see Supplementary Table S8) were detected in the off-field vegetation.

##### Vegetables

On average, 11 CUPs were extracted from vegetation inside the vegetable fields (mostly crop plants as wild plants were not available in large quantities due to herbicide use; see Supplementary Table S6). At one meter in the meadow, on average, 7 CUPs were detected, 7 at 5 m and 5 at 20 m (see Supplementary Table S6). CUP numbers decreased with the distance from the field (Fig. 2, Supplementary Table 5: b = − 0.22). Fungicides were detected most predominantly. The insecticide chlorantraniliprole was detected at all sampled distances (see Supplementary Table S8). In total, eight insecticides were recorded in-field, five at 1 m, two at 5 m and two at 20 m off-field.

##### Viticulture

On average, 12 CUPs were detected in vegetation from viticulture areas. The number of CUPs decreased slightly with increasing distance, with 10 CUPs recorded at 1 m, 10 at 5 m and 8 at 20 m (Fig. 2, see Supplementary Table S7: b = −0.15, see Supplementary Table S6). At the nature conservation area, a maximum of 16 CUPs was measured in July (May: 2 CUPs, June 7 CUPs, August 12 CUPs). No insecticide residues were detected in any vegetation sample from inside the vineyard or the adjacent meadows.

### Realistic mixture exposure risk assessment

The risks of single substances were calculated for the detected CUP mixtures and are summarised in the respective risk quotients.

**Table 2:** Overview of the mixture exposure risks (MRQs) calculated for collembola and earthworms based on the measured environmental concentrations (MECs) of 93 CUPs in topsoils in cultivated areas (in-field) of arable (*n* = 3), vegetable (*n* = 3) and viticulture sites (*n* = 3). The mean and maximum MRQ for both soil organisms at each agricultural site and month are shown. No risk (MRQ < 0.01, low risk (italics) 0.01 < MRQ < 0.1, medium risk (bold) 0.1 < MRQ < 1, a high (MRQ =1, bold italics) and very high risk (bold italics) MRQ > 1).

Month	Collembola						Earthworms					
	Arable site		Vegetable site		Viticulture site		Arable site		Vegetable site		Viticulture site	
	Mean RQT	Max RQT	Mean RQT	Max RQT	Mean RQT	Max RQT	Mean RQT	Max RQT	Mean RQT	Max RQT	Mean RQT	Max RQT
In-field (-20 m)												
Feb’21	**0.34**	**0.51**	**0.18**	**0.43**	*0.04*	*0.05*	**0.28**	**0.43**	**0.24**	**0.31**	**0.36**	**0.39**
Mar’21	**0.38**	**0.82**	**0.11**	**0.30**	*0.04*	*0.09*	**0.30**	**0.44**	**0.30**	**0.60**	***1.65***	***3.83***
Apr’21	**0.53**	**0.83**	**0.41**	***1.17***	**0.13**	**0.25**	**0.40**	**0.73**	**0.53**	**0.70**	**0.61**	***1.44***
May’21	**0.40**	**0.64**	**0.15**	**0.33**	**0.17**	**0.31**	**0.31**	**0.54**	**0.71**	***1.02***	***1.00***	***1.52***
Jun’21	**0.70**	***1.02***	**0.32**	**0.85**	***1.60***	***2.30***	**0.92**	***1.82***	***1.10***	***2.03***	***1.39***	***2.03***
Jul’21	***1.07***	***2.46***	**0.19**	**0.32**	***1.31***	***2.87***	**0.39**	**0.46**	***4.33***	***7.33***	***1.44***	***2.86***
Aug’21	**0.49**	***1.08***	**0.16**	**0.47**	***2.80***	***6.61***	**0.26**	**0.32**	**0.24**	**0.62**	***2.04***	***5.02***
Sep’21	**0.78**	***1.75***	**0.32**	**0.59**	**0.57**	**0.83**	**0.31**	**0.42**	**0.84**	***1.43***	***1.09***	***1.75***
Oct’21	**0.77**	***1.49***	***1.22***	***3.20***	**0.63**	***1.12***	**0.36**	**0.45**	***2.14***	***4.23***	***1.04***	***1.31***
Nov’21	**0.33**	**0.49**	**0.82**	***2.19***	**0.33**	**0.66**	**0.26**	**0.51**	***1.16***	***1.95***	**0.75**	***1.07***
Dec’21	**0.42**	**0.66**	**0.35**	**0.90**	**0.53**	**0.80**	**0.20**	**0.35**	**0.83**	***1.18***	***1.17***	***1.94***
Jan’22	**0.47**	**0.81**	**0.29**	**0.69**	**0.17**	**0.30**	**0.19**	**0.33**	**0.62**	**0.86**	**0.61**	**0.86**
Feb’22	**0.18**	**0.30**	**0.35**	**0.86**	*0.09*	**0.21**	**0.11**	**0.17**	**0.43**	***1.00***	**0.48**	**0.69**

#### Collembola

For Collembola, published no-observed effect concentration (NOEC) values were available for 55 of the 66 detected CUPs. In-field, CUP mixtures posed a risk (mixture exposure risk, MRQ) in all topsoil samples for collembola, a high risk was calculated in 13% (*n* = 5 of 39) of the samples (mean MRQ > 1), a medium risk (0.1 < MRQ < 1) in 79% (*n* = 31), a low risk ( 0.01 < MRQ < 0.1) in 8% (*n* = 3) and no negligible risk (MRQ < 0.01) was calculated (*n* = 0) (Table 2). High-risk values (mean MRQ) in-field were calculated in July for arable fields, in October for vegetable sites and from June to August for vineyards. The risk remained at a medium level at arable and vegetable sites but at a low level in February 2021, March 2021 and February 2022 at viticulture sites. In around 20% of the off-field samples, low (22%, *n* = 26) and medium (20%, *n* = 24) risks were calculated. A high off-field risk was calculated in the meadow soil at 1 m next to the vineyard in June (see Supplementary Table S9). The off-field environment posed a risk to collembola in approximately half of the samples (44%, *n* = 51 of 117; see Supplementary Table S9), whereby the occurrence of risks decreased with the increasing distance. On average the MRQ was highest on viticulture sites (mean arable sites (*n* = 156): 0.17, mean vegetable sites (*n* = 156): 0.11, mean viticulture sites (*n* = 156): 0.26).

The maximal Risk Quotient (RQ_max_), the risk caused by the concentration of a single CUP in the mixture, explained over all months 86% of the risk in arable in-field samples, 69% of the risk in vegetable in-field samples and 79% of the risk in vineyard in-field samples (see Supplementary Table S10). In a few instances also, single substance concentrations alone posed a high risk to collembola (RQ ≥ 1). The presence of four CUPs, cyflufenamid (*n* = 8 of 468 topsoil samples, six in-field and two samples at 1 m), epoxiconazole (*n* = 4, in-field), cyantraniliprole (*n* = 2, in-field) and imidacloprid (*n* = 2, in-field, see Supplementary Table S11), resulted in a high risk for collembola in the topsoil samples.

#### Earthworms

Toxicological endpoint values (NOEC, or if not available then LC50) for earthworms were available for 64 of the 66 CUPs detected in this study. The mean MRQ (*n* = 3) for in-field samples was calculated to indicate a risk for all months and all management systems. A high risk was calculated in 31% of the in-field samples (*n* = 12 of 39), a medium risk was calculated in 69% (*n* = 27) and no low or negligible risk was calculated (Table 2, see Supplementary Table S9). No high risk (mean MRQ) for earthworms was calculated in-field at arable sites. A high risk was posed by the CUP mixtures present in the topsoils of vegetable fields in June, July, October and November and for March, May to October and December in vineyards. The percentage of off-field mean topsoil samples at risk was 50% (*n* = 59 of 117), with 1% (*n* = 1) of all off-field samples at high risk, 23% (*n* = 28) at medium risk, and 25% (*n* = 30) at low risk (see Supplementary Table S9). The number of MRQs indicating a risk decreased with increasing distance. On average the MRQ was highest on viticulture sites (mean arable sites (*n* = 156): 0.11, mean vegetable sites (*n* = 156): 0.32, mean viticulture sites (*n* = 156): 0.34). Medium risks were detected up to 20 m in the meadow of the vegetable sites.

On average (*n* = 39), 46% of the MRQ is explained by a single CUP (RQ_max_) in in-field samples at arable sites and 62% in in-field vegetable and viticulture samples (see Supplementary Table S10). Four single CUPs posed a high risk to earthworms (RQ ≥ 1): the fungicides azoxystrobin (*n* = 2 of 468 samples), boscalid (*n* = 8), difenoconazole (*n* = 12) and the herbicide terbuthylazine (*n* = 1) (see Supplementary Table S12).

#### Wild bees

LD50 values for honey bees were published for 65 of the 66 CUPs detected in this study. The mean mixture hazard quotient (MHQ) for the acute exposure scenario (2.23 g soil exposure per bee in 48 h^36^) in-field posed a hazard in 20.5% of the topsoil samples (Table 3, see Supplementary Table S13). Based on the average MHQ (*n* = 3) a hazard (MHQ > 1) was inferred from June to July on arable land and from September to February 2022 on vegetable fields. No risk was identified at vineyards. The maximum MHQ indicated a hazard for wild bees from March to July on arable soils and in February and from August to February on vegetable soils. No maximum MHQ was higher than the threshold of 1 in vineyards (Table 3). Off-field, no hazard was calculated from the measured CUP mixtures (see Supplementary Table S13).

Over all months, 63% of the MHQ was explained by the concentration of a single CUP (HQ_max_) in arable in-field samples, 69% in vegetable in-field samples and 43% in viticulture in-field samples (see Supplementary Table S10). The concentrations of clothianidin (I, *n* = 4), cyantraniliprole (I, *n* = 7) and thiamethoxam (I, *n* = 3) exceeded the HQ threshold of 1 (see Supplementary Table S14).

**Table 3:** Mean mixture hazard quotient (MHQ) (*n* = 3) and the maximal MHQ were calculated based on the recorded concentrations of 93 CUPSs in in-field topsoil samples of arable, vegetable and viticulture site for solitary wild bees with a surrogate LD50 (honey bee LD50)/10^36^, with LD_50_ data taken from PPDB) and an acute contact of 2.23 g of soil over 48 h. The numbers displayed in bold are MHQs > 1, indicating a lethal hazard.

	Arable sites		Vegetable sites		Viticulture sites	
Month	Mean MHQ	Max MHQ	Mean MHQ	Max MHQ	Mean MHQ	Max MHQ
Feb’21	0.37	0.49	0.7	**2.08**	0.05	0.05
Mar’21	0.38	**1.13**	0.01	0.01	0.09	0.18
Apr’21	0.71	**1.8**	0.35	0.52	0.04	0.07
May’21	0.41	**1.19**	0.24	0.37	0.05	0.06
Jun’21	**1.75**	**3.9**	0.08	0.12	0.08	0.12
Jul’21	**1.54**	**4.54**	0.29	0.66	0.1	0.15
Aug’21	0.33	0.94	0.64	**1.91**	0.14	0.29
Sep’21	0.25	0.71	**2.18**	**5.65**	0.09	0.1
Oct’21	0.49	0.74	**7.87**	**22.69**	0.08	0.09
Nov’21	0.29	0.52	**5**	**14.82**	0.06	0.06
Dec’21	0.63	**1.28**	**1.32**	**3.93**	0.08	0.12
Jan’22	0.31	0.68	**1.4**	**3.64**	0.05	0.05
Feb’22	0.01	0.01	**1.55**	**4.09**	0.05	0.05

## Discussion

All topsoil samples in this study, in-field and off-field, contained at least one CUP, with an average of 10 CUPs per sample. This corresponds to other European studies, where at least one CUP was measured in 99%^2^ and 98%^3^ of the in-field topsoil sample. This suggests that soil in agricultural landscapes is contaminated with multiple CUPs. Methodologically, the number of detected CUPs depends on the number of CUPs that are analysed if targeted methods are used. For instance, in a French study, 111 different CUPs were included in a target analysis of topsoil samples, of which 67 different substances were detected^8^. We analysed 93 different CUPs and detected in total of 71 CUPs, 66 in topsoils and 62 in vegetation. In addition, Glyphosate and AMPA, were among the most frequently detected CUPs in the French study, with 83% and 70% detection rates, respectively^8^. These results correspond to a European-wide study^11^, which also detected a mixture containing AMPA and glyphosate (included in 25% of pesticide combinations^11^), to be the most common CUPs in soil samples. Therefore, we can assume that glyphosate and AMPA are very likely also occurring in our soils. In contrast, the most abundant CUP in our soil samples was fluopyram (in 94% of all topsoil samples), which was also detected in 69% of topsoil samples in a French study^8^ but was not included in the target substances in other studies^3,11^. Fluopyram is marketed as a fungicide in Europe but is used as a nematicide in tropical crops such as bananas^37^, therefore it has negative effects on nematodes and available data indicate a negative effect of a fluopyram metabolite on plants^38^ and soil arthropods^39^. Since our analysis covers only a part of the authorised CUPs in Germany, it is fair to assume that even more CUPs are present in in-field soils, resulting in even greater mixture complexity. We detected almost 350 different CUP mixture combinations in topsoil and vegetation by analysing 93 target compounds, with the most common mixture being detected only on eleven occasions in soils and seven in vegetation samples. The amount of combinations makes it impossible to assess the mixture toxicity risk organisms face in reality if this would ever be a regulatory aim. At least one insecticide was detected in 124 mixtures in soils and 68 mixtures in vegetation. In our target method, fewer insecticides than fungicides and herbicides are included, which may explain the lower frequency of insecticides observed in the mixtures. We assume that more mixtures contain insecticides than were observed in this study, as some insecticide groups, such as pyrethroids, can only be detected with different analytical methods. This limitation in detection capacity is an important caveat of our study, as it does not cover all authorised pesticides. To establish pesticide monitoring in the terrestrial environment, all registered molecules, as well as their metabolites or transition products, should be included in a target method and incorporated in the current proposal for an EU Directive on Soil Monitoring^13,40^.

A comparison of the detected substances with the application data provided by the farmers would have constituted an interesting research approach. However, as our study was conducted with actual farmers rather than model farms, we were dependent on their willingness to participate, which prevented the collection of such data.

Comparing cumulative concentrations across studies requires caution due to the use of different analytical methods, as underlined in a previous study^5^ and discussed above. Individual concentrations can be cautiously compared, considering variations in quantification limits, extraction methods and sampling times. It should also be considered that high variability in CUP concentrations has already been observed in soil samples^41^. Nonetheless, the highest concentration measured in our agricultural topsoil samples was detected for pendimethalin (981.18 µg/kg, vegetable, cultivated area, May 2021). In French arable soils, pendimethalin was detected at a concentration of 923 µg/kg in spring^5^ and at a concentration of 1,115 µg/kg in January^8^. In another study, a maximum concentration of pendimethalin (310 ng/g) was recorded after the field season^4^. The comparison shows that our data are quite comparable with those of other European studies when individual concentrations are considered.

The sampling time differed between studies; some were conducted before and others after the field season^42^. It was unclear whether the exposure data recorded in the studies were representative of the entire year or whether they were just displaying a specific moment. Our data show that a single sampling event is not representative of the whole year. Due to the differences in February 2021 and February 2022, there may likely be differences in exposure between years. In light of this, comprehensive, long-term monitoring is essential to gain a full understanding of the situation and its temporal dynamics. In the topsoils, we recorded a high baseline of CUPs throughout the year with various maxima. This result is not consistent with our hypothesis of seasonal variation, which was based on the timing of pesticide application. It might be influenced by multiple factors: CUP residues from deeper soil layers, which have been preserved there, can enter the topsoil through tillage. Crop senescence and harvest residues can also distribute CUP residues into the soil^43^. Moreover, the soil is also treated with CUPs before and after cultivation, mainly with herbicides to prepare the field for cultivation. During the agricultural season, the soil is covered by vegetation and most of the applied substances remain, depending on the development stage, on the crop foliage and only a small fraction reaches the in-field soil^44^. We therefore observed higher concentrations in vegetation than in soil. For example, the highest measured concentration in soils (pendimethalin, herbicide, 981.18 µg/kg) was orders of magnitude lower than the corresponding vegetation sample (23,161.44 µg/kg, vegetable site, in-field, May 2021), although the latter was not the highest concentration measured in vegetation. The highest concentration of an individual CUP in vegetation was recorded for dimethomorph (fungicide, 67,656.10 µg/kg, viticulture sites, in-field, July 2021), as the corresponding concentration in the topsoil was 6.86 µg/kg^34^. The maximal mean concentration for a single CUP was measured at 26.53 µg/kg metrafenone in topsoils and 1,349.55 µg/kg fludioxonil in vegetation, again showing the higher concentrations in vegetation.

To our knowledge, this is the first study investigating the annual variation of terrestrial CUP concentrations. To analyse our data an approach of loess smoothing and a Kullback-Leibler (KL)-divergence was chosen to capture the courses of the year as accurately as possible. By the use of usual complex models like for example GLM/GLMM limitations have quickly been reached with our data set, e.g. due to the unknown distribution of the data or the missing linear relation between the predictor and the explanatory variable. The chosen approach with loess smoothing and KL-divergence was also used in a recent ecology study where time series were tested^45^. In our analysis, we used the data from all three repetitions from one management system to model the general temporal trends, applying both a loess fit and an exponential function to the combined data. The loess fit smoothing over the repetitions, revealing the underlying temporal patterns^45,46^, while the exponential model is used in current pesticide emission models to estimate the drift^47,48^. Neither of these methods explicitly accounts for plot-specific variability, as they do not include random factors. CUP contamination in vegetation showed the hypothesised seasonal fluctuations, with an average of 7 CUPs per sample and a maximum of 25 CUPs in two single samples (in-field (−20 m), vegetable: celery, June and July). The observed fluctuations in the loess fit indicate a substantial deviation from the surrounding months. Although the Loess method does not provide p-values or formal statistical significance, the robustness of the fit implies that this peak reflects a meaningful difference, rather than random variation^49^. Grass samples from public areas in the Vinschgau Valley taken four times per year showed statistically significant changes in CUP contamination between seasons^9^. The highest CUP numbers were detected in early summer (May/June), while we measured maxima in late summer (July and August). The Vinschgau Valley is dominated by apple orchards that are treated earlier than the crops that we studied. However, fungicides were the dominant group in both studies.

Vegetation, both in-field and off-field, is more likely than soil to be contaminated by wet deposition and spray drift^50^, as soil is covered for a long period by vegetation. Additionally, vegetation takes up systemic CUPs from the soil^51^, which may account for some of the CUPs that were measured at lower concentrations. However, dilution effects caused by plant growth, as well as off-field mowing and grassing, can strongly influence the concentrations measured in vegetation samples. These factors present inherent challenges in field experiments, as they are difficult to quantify and cannot be easily accounted for in statistical analyses.

CUP residues were detected in the majority of the samples throughout the year. We show that the soils of agricultural areas, as well as those of the immediate surroundings, are loaded with mixtures of different CUPs throughout the year, indicating potential chronic CUP exposure for insects. Nevertheless, we also detected maxima and minima for the number of CUPs (Supplementary Tables 5, 8). Most maxima were measured in autumn, which can be explained by the accumulation of CUPs throughout the field season.

It is still unclear what effects the presence of up to 28 different CUPs (measured in in-field soil in arable field in October) has on insects and other organisms^52^. Although meadows were less contaminated than cultivated, in-field areas, mixtures of multiple CUPs were detected all year round.

We assumed that the number of detected CUPs decreased with increasing distance to the field. The decrease was tested by exponential functions, commonly used to model pesticide drift (e.g^53^). The hypothesised decrease in CUP numbers was detected for topsoil samples for all three management systems but less so for vegetation samples. In the latter, similar numbers of CUPs were recorded at different distances in the meadow. The number of CUPs was highest in the meadows adjacent to the vineyards and was less different from the in-field values than for the other management systems. This might be explained by the different ways of application; the 3D application, which is used for room cultures like viticulture or in orchards, to cover the canopy, leads to higher drift effects^54^. This finding is consistent with our hypothesis that there are differences between the various application types.

While the study design includes three repetitions per management system, with a total of 18 plots (9 in-field and 9 off-field), we acknowledge that this level of replication may still be considered limited, particularly in the context of the high variability between sites and management systems. The relatively low number of repetitions could make it challenging to fully account for all sources of variation. However, this design was a balance between practical constraints and the need to capture variability across different conditions. Conducting larger-scale studies with more repetitions would have been ideal, but such an approach is often constrained by logistical factors. Future studies could benefit from increasing the number of repetitions or expanding the range of covariates considered in the analysis to better capture the complexity of the system. On the other hand, we collected a large, unique dataset of soil and vegetation samples, comprising approximately 1,000 samples over the course of a year. In this field of research studies so far comprised e.g. 75 soil samples in Hvězdová et al. 2018^2^, 317 soils in Silva et al. 2019^11^, 180 soils in Pelosi et al. 2021^5^, 71 vegetation samples in Linhart et al. 2019^7^ or 96 vegetation samples in Linhart et al. 2021^9^. Our comprehensive dataset has the potential to benefit other researchers in a number of ways: It can be used as a benchmark for comparative studies, as a resource for secondary analyses, or as a foundation for model development and long-term monitoring efforts.

In all soil samples (*n* = 468), CUPs were detected, while in 14 of 442 vegetation samples, CUPs were not detected. Most uncontaminated samples were recorded at the greatest distance from the field in 2 D crops (20 m off-field: *n* = 7 of 14 and 5 m *n* = 6). In a study investigating CUP residues in vegetation at similar distances, no CUPs were detected in 5 of 7 vegetation samples at their furthest distance (> 10 m), in 2 of 8 at 5–10 m, and all field edge samples (9 samples) were contaminated^32^. In a landscape characterised by agriculture, CUPs are recorded not only at the edge of the agricultural field but also at further distances, as shown here and also in previous studies^7–10,13,32,55^.

Insects that live in an agricultural landscape are therefore potentially exposed to CUP mixtures not only by visiting or living in the fields themselves but also in the surrounding area, e.g. adjacent meadows. We assume that the main source of CUP contamination in vegetation samples from the meadow was drift during applications^10,56^ or via volatilisation and/or dust from soil and plants after application, due to the physical-chemical properties of the pesticides, which are known to facilitate such processes (e.g., volatility, adsorption to particles). The pesticide concentrations in vegetation from public playgrounds were already positively correlated with the amount of rainfall and wind speed during application times^7^. However, air deposition^57^ and rain events^58^ are also inputs of CUPs, with transport from sources several kilometers away.

In our study, we also addressed the risk of CUPs to insects. However, the publicly available toxicology data basis is not sufficient, as there are no toxic endpoints for soil insects. Furthermore, as there is no risk assessment for herbivorous insects (e.g. grasshoppers or caterpillars) required, there are no toxic endpoints for oral insect exposure. Therefore, no risk from the consumption of contaminated vegetation could be calculated. Nevertheless, for approximately half of the CUPs detected in topsoils, the concentrations were below 1 µg/kg, while the concentrations for individual CUPs in vegetation samples were higher (7 of 61 CUPs with concentrations below 1 µg/kg). Concentrations in vegetation samples reached up to 67,656.10 µg/kg (dimethomorph, F, viticulture sites, in-field, July 2021). While insecticides were not detected at all off-field distances in topsoil, they were detected at all distances in vegetation samples from arable and adjacent meadows, indicating that the risk to insects is likely to be even higher here. These findings highlight the need to develop and analyse risk approaches for oral uptake via vegetation.

We showed that, on average, soils from the field posed a high to medium risk throughout the year for earthworms and collembola, except in three months for collembola (mean MRQ, Viticulture, February 2021, March 2021, February 2022, low risk). Furthermore, we showed that samples from meadows also posed a risk to collembola and earthworms, although the risk decreased with increasing distance to the agricultural field for all land use types. However, as nontarget areas also harbour chronic risks for at least earthworms^5,8^, potential exposure-free and low-risk habitats for organisms are becoming increasingly scarce. As toxicological endpoints for springtails were not available for all measured CUPs, the risk might be even higher. The bioavailability of residues and the potential for synergistic effects of CUP mixtures need to be further investigated. Recently, a meta-analysis showed that the risk of pesticide exposure, assuming additive effects, underestimates the interaction effect on bee mortality, which in most cases is synergistic^59^. In our study the in-field risk was to some extent explained by single CUP concentrations, explaining on average 43 to 86% of the mixture risks, depending on the management system. Also, some single CUPs represented a high risk. The reduction or even avoidance of CUPs that trigger a high risk might improve the risk situation for non-target soil organisms. We identified eleven fungicides (azoxystrobin, boscalid, cyflufenamid, difenoconazole, dimoxystrobin, epoxiconazole, fluopicolide, fluopyram, metrafenone, myclobutanil, penconazole) five herbicides (clomazone, ethofumesate, pendimethalin, propyzamide, terbuthylazine) and five insecticides (chlorantraniliprole, clothianidin, cyantraniliprole, imidacloprid, indoxacarb) that contribute substantially to the measured risks. However, by changing CUP applications and substances the risk of other substances might increase.

There is currently no approach that addresses pesticide residues in soils as an exposure route for insects. Most wild bees nest in the soil^25,60^. Ground-nesting bees are in direct contact with the soil and larvae are exposed not only to the residues in the soil but also to the pollen. An in-field assessment of the risk to ground-nesting bees from systemic insecticides was recently performed^36^. Due to a lack of information, the authors used toxicological endpoints for honeybees to determine the risk for adult females to experience soil contact during nest building^61^. The ground-nesting honey squash bee (*Peponapis pruinosa*) used in this approach does not exist in Europe, but several other ground-nesting bees are known to build similar nests in agricultural soils in Europe^62^. *Peponapis pruinosa*, it is similar in size to common ground-nesting bees such as *Andrena flavipes*, which have been observed building nests in European agricultural fields^62^. 

Our wild bee risk evaluation shows that in-field, the CUP residues lead to an MHQ > 1 in arable and vegetable fields but not in viticulture. This may be because insecticides are not or less commonly used in the latter. At the vegetable sites, an MHQ > 1 was calculated from August to February, when most of the ground-nesting adult bees were no longer active. However, larvae and pupae overwinter in soils and are therefore assumed to be continuously exposed to potential pesticide residues, although their dormant phase within cocoons may on the other hand reduce their direct exposure generally uptake of pesticides can currently only be assumed as no data are available. We consider this aspect of the risk assessment as a supplementary evaluation of the potential exposure of soil-living insects and their development stages. The calculated risk may not lead to direct mortality in organisms but sublethal effects that occur at lower concentrations result in population-level effects e.g. due to lower reproduction^63^, especially with chronic exposure over longer time spans.

We detected different insecticides in topsoils and vegetation at all sites and in vegetation at arable and vegetable sites. Chlorantraniliprole (I) was the most commonly recorded insecticide in both matrices. It is a newer insecticide used for tree fruits, grapes, vegetables, and maize^64^. Chlorantraniliprole reduced the locomotor activity of honey bees after 72 h of exposure^65^ and was already detected in honey bee pollen^66^, supporting our results. Pendimethalin, which together with terbuthylazine was the most frequently reported herbicide in topsoil samples, can affect the immune response and reduce the lifespan of honeybees^67^. The herbicide with the most detections in vegetation, prosulfocarb, was also detected with the highest frequency in German urban and agrarian bumblebee samples^68^. Prosulfocarb (H) and terbuthylazine (H), were also detected in all insect samples from 21 nature conservation areas in Germany, as were the fungicides fluopyram and azoxystrobin^52^. These results are consistent with our findings that insects are particularly frequently exposed to these substances.

Current risk assessments are based on the effects of single substances on individual organisms. Furthermore, it is assumed that there is sufficient time after application for populations to recover to previous levels^22^. However, our results show that there are no CUP-free periods. It is therefore improbable that recovery can be achieved in a realistic situation. In particular, in the field soil, high numbers of CUPs are recorded all year round, resulting in chronic exposure of soil insects or life stages of insects, e.g., larvae or pupae. High-resolution sampling over a year enables us to obtain CUP data about the activity of insects. In the vegetation, there is a maximum amount of CUP detected in the summer months, when many insect larvae feed on the plant material (e.g. Lepidoptera larvae) or use the vegetation for oviposition. When exposed to CUP residues as larvae on host plants, effects on longevity and wing length were observed in emerging butterflies^69^. Insecticide-treated plants were found to experience reduced pollination and limited to a single egg-laying event by moths, resulting in a 40% decrease in their offspring^70^. For adult wild bees (*Osmia cornuta*) exposed to sublethal concentrations of a mixture containing only one fungicide and one insecticide, a reduction in reproduction rate was observed, ultimately leading to a decline in population size^71,72^. Furthermore, insecticides have been shown to have carryover effects on wild bees^73^ and other nontarget insects^74^.

## Conclusion

This investigation provides to the best of our knowledge the first temporal resolution of CUP residues in topsoil and vegetation in different management systems and adjacent non-target areas as common structural features in agricultural landscapes. In particular, complex mixtures of multiple CUPs were present throughout the year. They were not only recorded in the cropping area (in-field) but also in neighbouring, non-target meadows at distances of up to 20 m. This constant presence of multiple CUPs in agricultural landscapes leads to a chronic mixture of exposure to insects and other non-target terrestrial organisms.

The documented CUP exposure pattern is particularly worrying considering that most of the adult insects are mainly active in summer when the greatest number of CUPs are detected in vegetation. It has already been shown that flying insects collected in nature conservation areas are exposed to multiple CUPs^52^. In the topsoil, on the other hand, a high number of CUPs are present throughout the year, so adult insects or larvae living in the soil are chronically exposed to several CUPs at low concentrations. It is important to note that the constant presence of CUP mixtures is not part of the regulatory environmental risk assessment procedures for pesticide regulation. The remit of authorities at the EU and national levels lies in the risk characterisation of single substances and only occasionally are mixtures addressed in formulated products with up to four CUPs. We therefore urge authorities to ensure that chronic contamination with complex CUP mixtures occurring in reality is incorporated into authorization procedures and risk assessments, to prevent an unprotected environment. While risk management and mitigation strategies exist, pesticides are nevertheless detected even in areas far removed from agricultural landscapes^55^.

Furthermore, together with the demonstrated landscape-scale distribution of CUP mixtures during the application time^13^, our analysis demonstrates that this large-scale contamination is to be expected throughout the year. Additionally, the complexity of mixtures and the calculated risks are supposed to be even higher, as our analysis included only a fraction of the CUPs used in Europe (93 of the most commonly used out of 281). In the context of the observed insect declines, it is crucial to recognise, identify and reduce the risks posed by CUPs to insects. As mixture exposure is not addressed in the EU regulation, only reductions in pesticide risk, as included in national targets in the EU (e.g. Germany’s National Action Plan aiming to minimize the risk associated with pesticide use^75^, or France’s commitment to reducing the use and risks of pesticides^76^) or globally by the UN until 2030^77^, can change the currently observed declines. Together with conversion targets to reach organic practices in 25% of the agricultural area^78^, where no synthetic pesticides are used, the required transformation of agriculture is started and exposure of organisms reduced, allowing the recovery of ecosystem services for a truly sustainable agriculture.

## Materials and methods

### Study sites

**Fig. 3:**
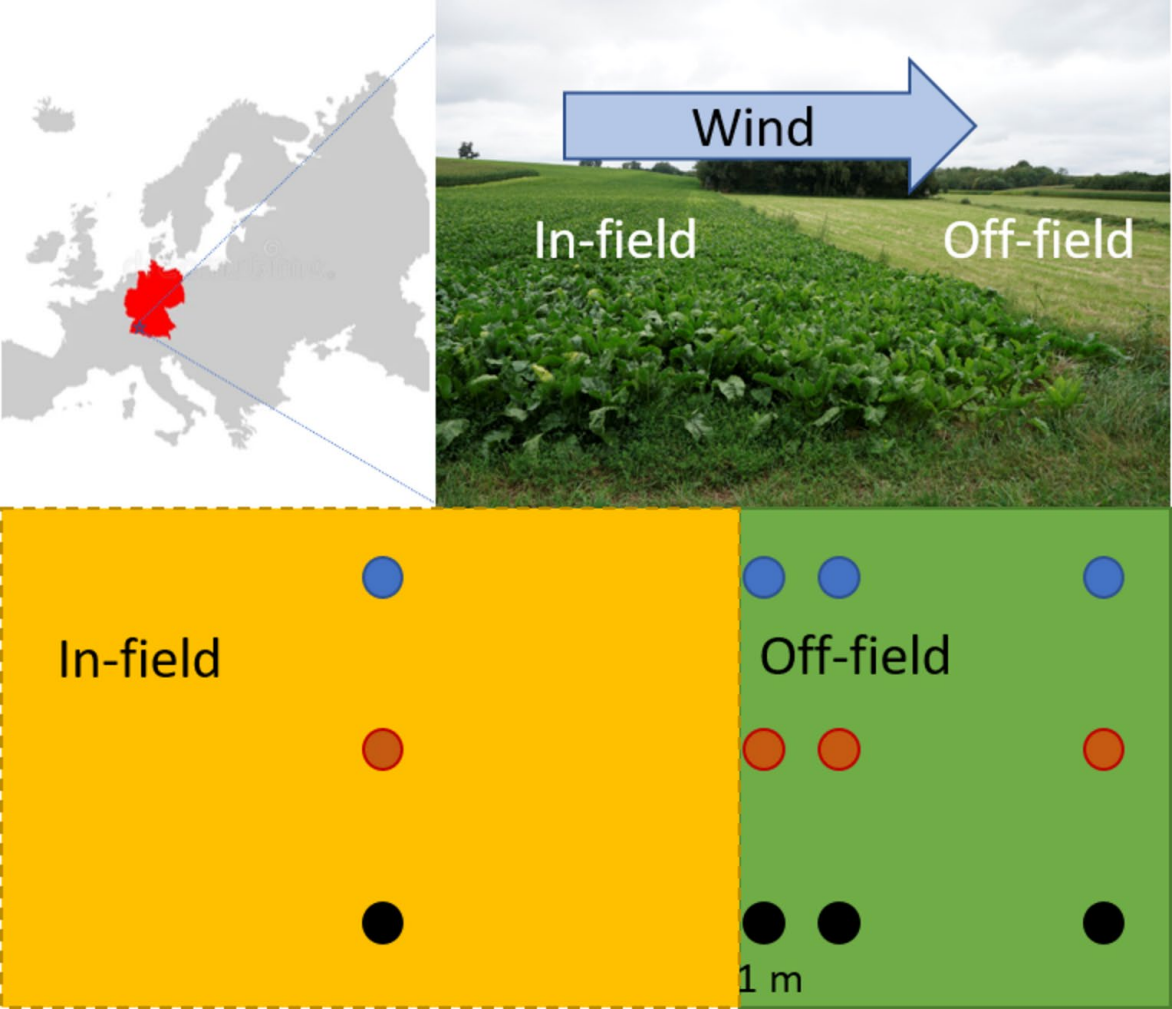
Study area and layout of the field and meadow: The study was conducted in the southern Rhineland-Palatinate (blue star) in Germany between Bad Bergzabern (district Südliche Weinstraße) and Schwegenheim (district Germersheim). On each of the nine sites, a field and an adjacent meadow were studied. Soil and vegetation samples were taken 20 m from the field border in the cultivated area (field, yellow square) and at three distances (1, 5 and 20 m from the field border into the meadow) in the meadow (green square). For each sample three subsamples (blue, red and black dots) were taken with a 5-meter distance between them, leading to four samples per month and sample site. Samples were taken monthly from February 2021 to February 2022.

This study was conducted on cultivated areas and meadows downwind of the main wind direction (SW) in southern Rhineland-Palatinate in Germany (Fig. 3). Sampling occurred monthly between February 2021 and February 2022 (see Supplementary Table S15), to cover a whole year, including the most important application periods, but also the seasons with less intensive pesticide applications. The study included nine fields with each having an adjacent meadow. Conducting fieldwork across a wider range of sites was not feasible because sampling required visits every four weeks, involving considerable travel time between locations. Ensuring consistent, high-quality sampling within the available resources was prioritized over increasing the number of fields. Furthermore, the process of recruiting participating farmers was challenging, as only a limited number were willing to contribute to the study. Requesting detailed pesticide application records on top of this could have further discouraged participation. Consequently, the study focused on assessing broader residue patterns rather than site-specific application regimes, balancing practical limitations with research objectives. Our farms were also not part of a model farm network and therefore their applications reflect a common and realistic conventional pesticide management.

We chose three fields for each management system (arable farming, vegetable cultivation, vineyards). We investigated three fields for the arable managing system representing crops typical of the Rhenish crop rotation system^79^: one field with a cereal crop (winter wheat) and two with leafy crops (maize, sugar beet). This selection reflects the sequences in the cropping system. While the replication at the field level was limited, the fields were chosen to represent different cropping practices commonly observed in the region. The vegetable fields followed a succession planting pattern (e.g., fennel followed by celery, then radish, Supplementary Table 16). Although multiple fields of the same crop per management system, such as wheat, would have been preferable to reduce variability, the selection was constrained by the fields that were available for inclusion in the study. Given the limited number of fields accessible, which included a mix of crops for arable farming and vegetable cultivation, the study aimed to reflect the diversity of agricultural practices encountered in real-world conditions and to capture variations in agricultural practices and pesticide application methods. Arable farming typically involves long-term crops that remain in the field for extended periods, with pesticides applied in a two-dimensional (2D) spraying. In contrast, vegetable cultivation is characterised by quicker crop rotations, also involving 2D pesticide applications. Vineyards, on the other hand, involve a permanent crop, with pesticide applications occurring in three dimensions (3D).

We included an adjacent meadow to each field as a non-target area for comparison. All the farms followed conventional management practices involving the use of synthetic pesticides. Pesticides were applied to the fields according to good agricultural practices based on the specific crop. Six of the nine meadows were used for long-term hay production, two others were privately owned or municipal long-term green spaces and one was a nature conservation area. All meadows were only mowed once or twice during the year and consisted mainly of grasses. The sampling was conducted with the explicit permission of the landowners. All landowners were informed, that the information they provided is used for publication. In order to ensure that landowners remain anonymous, no further details of the exact locations are given.

### Sampling and sample preparation

Sampling was carried out at each of the nine sites, at four sampling points each (20 m in the cultivated field (in-field, target area), 1 m, 5 m and 20 m from the field edge into the meadow (off-field, non-target area), Fig. 3). Samples were taken at all sites (*n* = 9) and distances (*n* = 4) each month (*n* = 13). At all distances and months, it was possible to take soil samples, resulting in 468 topsoil samples, while not at all in-field samples vegetation was growing at all months, resulting in a total of 442 vegetation samples. For each sample, a composite sample was obtained from three subsamples collected at a distance of 5 m from each other (Fig. 3) to get representative samples.

For the soil samples, after removing the vegetation and root layer, 0–5 cm topsoil was taken with a flower onion planter tool (Gardena, Ø 12.5 cm) at three locations per distance and stored in a sample bag combining the three subsamples. The topsoil samples were weighed in the field, transported to the laboratory and stored in a freezer (−20 °C). The samples were then freeze-dried for 48 h (Alpha 1–4 LSCbasic, Christ, Osterode, Germany), sieved (2 mm, standard-compliant test sieve, ISO 3310-1, Haver & Boecker OHG, Oelde, Germany) and homogenised. For the extraction of CUPs from topsoils, 5.00 g ± 0.01 g of soil material was weighed (ME802, Mettler Toledo, Ohio, USA; d = 0.01 g).

For the vegetation samples, cultivated plants and “weeds” (if available) were collected in the field. For the off-field samples, differing grasses and herbaceous plants were collected in the meadow. The samples were hand collected at three locations (see above) using laboratory gloves, filling half of a 1 L freezer bag with the three subsamples. The above-ground vegetation (approximately upper 20 centimetres) was taken as vegetation samples. The combined vegetation samples were first air-dried overnight in the laboratory at room temperature, frozen (−20 °C) and subsequently freeze-dried for 24–48 h, depending on the moisture of the sample. The vegetation samples were ground in a knife mill (Grindomix GM 300, Retsch, Haan, Germany) for 2 × 1 min at 3000 rpm to avoid heating. The ground vegetation was stored in small freezer bags at −20 °C until extraction. For extraction, 1 g ± 0.01 g of vegetation powder was weighed into 50 ml tubes. The reason for using less matrix for vegetation samples compared to soil samples is primarily due to the availability and volume of material collected during the sampling process. Nonetheless, the precision is the same with also a low error margin for vegetation samples. The relative error of about 1% for vegetation would not significantly influence the CUP concentrations and can be neglected.

All tools used in the field were cleaned with ethanol (≥ 98%, Carl Roth GmbH + Co. KG, Karlsruhe, Germany) and tap water e.g. the onion planter tool and in the lab with acetone (≥ 99.9%, Carl Roth GmbH + Co. KG, Karlsruhe, Germany) and tap water. All other materials e.g. sieves were also cleaned with acetone and tap water.

### Analytics for CUP residues

Validated multicomponent analytical methods^80^ were used to extract 93 CUPs (36 fungicides, 36 herbicides, 21 insecticides) from soil and vegetation. Details on the CUP selection can be found in^80^, the most frequently-used herbicides and fungicides as well as all insecticides from 2016 to 2017 recorded by the Julius-Kühn Institute were selected^81^. By 2021, three fungicides, five herbicides and eight insecticides had been withdrawn from the market, in total 77 of the 93 substances were approved in 2021. In brief, 5.00 g of soil or 2.00 g of vegetation was extracted with 10 ml of acetonitrile (MeCN, HPLC gradient grade ≥ 99.9%, Honeywell, Charlotte, USA) containing 2.5% formic acid (CH_2_O_2_, HiPerSolv Chromanorm ≥ 99.0%, VWR, Radnor, USA) and either 5.00 g–2.00 g of salt (ammonium formate, NH_4_HCO_2_, reagent grade ≥ 99.0%, Sigma‒Aldrich, St. Louis, USA) for soil and vegetation, respectively. The samples were placed in an overhead shaker for 1 h (Stuart drive Rotator drive STR4, Cole-Parmer, Vernon Hills, USA); afterwards, the samples were centrifuged (MegaStar 1.6R, VWR, Radnor, USA, 6 min at 3000 rpm) and filtered (17 mm HPLC syringe filter, PTFE, pore size 0.2 μm; La-Pha-Pack, Langerwehe, Germany). The vegetation extracts were further cleaned with graphitised carbon black (GCB, Carbon SPE Bulk Sorbent, Agilent Technologies, Santa Clara, USA). For the clean-up, 1 ml of vegetation extract was added to 7.5 mg of GCB in a 2 ml Eppendorf tube. The tubes were vortexed vigorously for 60 s and the supernatant was filtered through 2 μm filters directly into the measurement vials.

The extracts were analysed by high-performance liquid chromatography-tandem to triple quadrupole mass spectrometry via electrospray ionisation (HPLC)-ESI-MS/MS (HPLC: Agilent Technologies LC 1260 Infinity II series, MS/MS: Agilent Technologies 6495 C, Santa Clara CA, USA). The instrumental performance and potential carry-over effects were controlled by injecting quality control and analytical blank samples, respectively. For quality control, all the samples were spiked with 50 µL of the deuterated standard imidacloprid-D4 (98.9%, Dr. Ehrenstorfer GmbH, Augsburg, Germany) in acetonitrile (MeCN, HPLC gradient grade ≥ 99.9%, Honeywell, Charlotte, USA) (concentration: 10 mg/L). The solvent was allowed to evaporate for 30 min under a fume hood. A blank sample was spiked with imidacloprid-D4 after extraction (concentration: 10 mg/L). The peak areas of imidacloprid-D4 in the samples and of the respective blank samples were compared to check for extraction quality.

HPLC-ESI-MS/MS data were processed with Agilent Mass Hunter Quantitative Analysis software (Version 10.0., Build 10.0.707.0, Agilent Technologies, 2006–2018, Santa Clara CA, USA). CUP residues were considered positive if the MRM-transition products were present and if the retention time, response and quantifier/qualifier ratio corresponded to the standard sample at a comparable concentration (range: 70–130%). The quantification of CUP concentrations in all samples was performed using external matrix-matched calibrations. Detailed information on the limit of quantification (LOQ) and limit of detection (LOD) is available at^80^. For the total number of detected CUPs in the samples, residues < LOQ were also included. However, for the calculation of total CUP concentrations, only residues > LOQ were used.

### Risk assessment

We used the measured topsoil CUP concentrations to calculate a conservative cumulative chronic risk for earthworms and collembola, as well as a hazard quotient for wild bees. For these calculations, only soil contact exposure was considered, as no other exposure routes were included. To date, there are no recommendations for approaches that include potential synergistic effects^5^, so additive approaches were chosen. For HQ calculations, we used an assessment factor of 10 for comparability with other recent studies^5,8,12,82^. The Hazard Quotient (HQ) approach was used to assess the risk of ground-nesting solitary bees^36^. There is limited availability of toxicological endpoints for insects and approved risk assessment approaches. We chose the proposed approach to determine the risk posed to ground-nesting insects from soil based on the concentrations detected. The honey bee lethal effect endpoints (median lethal dose, LD_50_) were used because honey bees are the current regulatory standard and are considered to be an appropriate proxy for all bee species^83^. We used the contact acute LD_50_ values reported in the “Pesticides Properties DataBase” (PPDB^84^). A surrogate solitary bee contact LD_50_ (honey bee LD_50_/10) was also used. The matrix of a ground-nesting bee (*Peponapis pruinosa*) was exposed as described previously^36^. This wild bee does not occur in Europa. However, other wild bees such as *Andrena flavipes*, *Halictus scabiosae* or *Systropha planidens* also nest in soil, are similar in size and occur in or near agricultural areas in Europe^85,86^. We estimate a similar exposure to * Andrena flavipes *whose nests are similar to *Peponapos pruinosa* which is common in Europe and was detected in European agriculture fields^62^.

It was estimated that female *Peponapis pruinosa* bees have contact with 2.23 g of soil during their burrowing activity for nest construction during one day^36^. Only soil contact exposure was addressed in the calculation.

The HQ was then calculated for each CUP residue in each topsoil sample as follows:

1 $$HQ= \frac{\left(Matrix\, exposure\, \left[g\right]\right) \left(MEC\,\left[\frac{ng}{g}\right]\right)}{\frac{{honey\, bee\, LD}_{50}}{10}\left[\mu \frac{g}{bee}\right]})=\frac{\left(2.23\, g\right) \left(MEC\,\left[\frac{ng}{g}\right]\right)}{\frac{{honey\, bee LD}_{50}}{10}\left[\mu \frac{g}{bee}\right]})$$

Individual HQ values from one topsoil sample were further summed to obtain the mixture hazard quotient (MHQ, Eq. 2) in the topsoil sample.

2 $$MHQ=\sum HQ$$

An HQ > 1 indicates a potential lethal hazard occurring from the CUP residues. The concentration of residues was given in µg of active ingredient (a.i.) per kg of soil matrix, which equals ng (a.i.)/g (matrix); the amount of exposure to the matrix was reported in g of matrix per bee and the LD50 values were reported in ng a.i. per bee.

Additionally, the risk posed by cumulative concentrations of CUPs for earthworms was calculated following the methodology proposed by^12^ and applied in recent studies^5,8,12,82,87^. The chronic no-observed-effect concentration (NOEC) for earthworms (*Eisenia fetida*) and collembola (*Folsomia candida*) from the PPDB were utilised. If a no-observed effect concentration (NOEC) was not available, reports to the European Commission and reports of the European Food and Safety Authority were used. If no NOEC was available, then the lethal concentration where 50% are killed (LC50) was used. The toxicity assessment for earthworms was based on 55 NOECs and 9 LC50 values, while for collembola, all were based on the NOEC (*n* = 55; see Supplementary Material Table S18). The NOEC was divided by an assessment factor of 10^5,8^, or the LC50 by an assessment factor of 1000^5^, to compute the predicted no-effect concentration (PNEC)^88^. The risk quotient (RQ) was then individually calculated for each CUP as the difference between the PNEC and the measured environmental concentration (MEC) in each soil sample (Eq. 3, SI, Eq. (1) to (7)).

3 $$RQ=MEC/PNEC=MEC/(NOEC/AF)$$

Subsequently, the mixture exposure risk (MRQ, Eq. 4) was calculated for each topsoil sample.

4 $$MRQ=\sum RQ$$

The MRQs were further categorised into four risk groups: negligible risk, MRQ < 0.01; low risk, 0.01 < MRQ < 0.1; medium risk, 0.1 < MRQ < 1; high risk, MRQ = 1, very high risk ≥ 1^89^. The same procedure was used for the data from the springtail *Folsomia candida*.

### Statistical analysis

For the statistical analysis, Rx64 4.0.1 was used^90^. For samples with values below the LOQ, the concentration was set to 0 for statistical analyses. To avoid multicollinearity issues, principal component analyses (PCAs) (package: stats, function: princomp^90^) were constructed. To identify local CUP minima and maxima during the year, a linear model was built to subtract the trend for the number of CUPs of the year from the number of shorter-term fluctuations; furthermore, a peak detection algorithm was used^91,92^. This enables us to compare CUP residues and insect development stages. The algorithm used a threshold delta value to determine when a peak (maximum or minimum) was reached. The algorithm iterates through a given vector, updating the maximum or minimum candidate based on the current value and the threshold. If a current value deviated by more than a delta from a previous extremum, a new extremum was detected. When a peak was identified, its position and value were recorded. To increase the overall detection rate, the delta was set to 0.3. A delta of 0.3 was considered appropriate as it allows for the detection of a broader range of relevant signals without significantly compromising the reliability of the results. We acknowledge that it is somewhat subjective. In general, delta values are often set based on the dataset, to increase the overall peak detection rate (e.g. in^93^). The peak finder is only used to standardize the interpretation of the peaks and does not influence the measured and fitted peaks themselves. We constructed a generalised linear model (GLM) to see, whether the predictors have a significant influence on the number of CUPs (see SI GLM section). For the analyses of the courses over the year, the following approach was used, which has recently been applied to quantify differences in time series patterns across ecological systems^45^: A loess smooth was used to obtain a coherent progression over the year and distance to the field margin^49^. The loess smoothing was further cross-validated, for further details on the loess smoothing, see the SI Loess smoothing section. Individual loess smooths were compared with the Kullback-Leibler (KL) divergence. KL-divergence is used to measure differences in distributions or patterns^45,94^. It can be considered as a measurement of information^95^.

The KL divergence was calculated as follows:

5 $$KL\left(P\|Q\right)=\sum P\left(P\right)\text{*}\text{log}2\left(P\left(P\right)/P\left(Q\right)\right)=H\left(P,Q\right)-H\left(P\right)$$

where $H(P,Q)$ denotes the joint entropy of the probability distributions $P$ and $Q$ and $H\left(P\right)$ denotes the entropy of probability distribution $P$^96^. KL divergence, while using direct yearly progressions, allows a comparison of curve patterns (for the SI, see the KL divergence section).

The distance to the field margin was fitted with an exponential function with nlsLM from the minpack.lm package^35^. It was assumed that the measurements in cultivated areas (−20 m) also represented the concentration at the field margin (0 m). This assumption was made because the −20 m measurement is considered representative of the general in-field concentration. Measurements at the field border (0 m) would likely not be fully representative, as the field edges are often subject to lower pesticide rates due to drift reduction techniques, which aim to minimize off-target drift. Rather than interpreting the 0 m measurement as the CUPs at the field edge, it is more accurate to consider it as indicative of the in-field measurement.

The exponential function was set up as:

6 $$y=a * {e}^{\left(b * x\right)}+c$$

where $a$ is the amplitude, $b$ is the base, c is the constant term, $y$ is the response variable and $x$ is the explanatory variable. In this study, the value $a$ served as a vertical scaling factor, describing the amount of additional CUP detected at the margin in comparison to higher distances. The value $b$ indicates the decay speed (if negative). Lower $b$ values represent faster decay. The mean of the respective subset of the − 20 m distance was used for $c$, describing the background concentration that the function should have reached.

## Electronic supplementary material

Below is the link to the electronic supplementary material.

Supplementary Material 1

Supplementary Material 2

Supplementary Material 3

Supplementary Material 4

Supplementary Material 5

Supplementary Material 6

Supplementary Material 7

## Data Availability

Data is provided within the manuscript, the supplementary information and under https://doi.org/10.5281/zenodo.11517560.
