# Supplementary material for: Exposure of insects to current use pesticide residues in soil and vegetation along spatial and temporal distribution in agricultural sites
Source: Sci Rep. 2025 Jan 21;15:1817. doi: 10.1038/s41598-024-84811-4 (PMC11751026; doi:10.1038/s41598-024-84811-4)
Supplement: Supplementary file 6 — Supplementary Material 6 [file 41598_2024_84811_MOESM6_ESM.docx]

Supplementary Information

# **Exposure of Insects to Current Use Pesticide Residues in Soil and Vegetation along Spatial and Temporal Distribution in Agricultural Sites**

Carolina Honert^1*^, Ken Mauser^1^, Ursel Jäger^1^, Carsten A. Brühl^1^

*^1^ iES Landau, Institute for Environmental Sciences, University of Kaiserslautern-Landau, Fortstraße 7, 76829 Landau, Germany*

*Corresponding author: Carolina.honert*@*rptu.de*

**Tables**

Table S1 Overview of number and frequency of detected CUPs, the mean concentrations and the maximal detected concentrations in 468 soil and 442 vegetation samples. Empty cells mean no detections. Top 3 values per matrix, pesticide group and rubric (detection frequency, total mean concentration and maximum concentrations) are coloured in red. Substances which were not approved in 2021 are marked with an asterisk (*).

|  | **Detection Frequency [%]** | | **Total Mean Conc. [µg/kg]** | | **Maximum Conc. [µg/kg]** | |
| --- | --- | --- | --- | --- | --- | --- |
|  | Soil  (n=468) | Vegetation (n=442) | Soil  (n=468) | Vegetation (n=442) | Soil  (n=468) | Vegetation (n=442) |
| **Fungicides** |  |  |  |  |  |  |
| Azoxystrobin | 57.91 | 35.97 | 6.02 | 306.15 | 525.92 | 22704.56 |
| Benalaxyl | 15.81 | 23.3 | 2.88 | 14.97 | 86.63 | 576.29 |
| Bixafen | 14.32 | 0.45 | 2.72 | 1.2 | 15.06 | 1.49 |
| Boscalid | 76.07 | 37.56 | 15.79 | 240.68 | 394.35 | 9220.43 |
| Cyazofamid | 7.69 | 24.66 | 1.95 | 396.27 | 35.99 | 6243.01 |
| Cyflufenamid | 16.03 | 38.91 | 1.04 | 14.52 | 11.25 | 298.74 |
| Cymoxanil |  |  |  |  |  |  |
| Cyprodinil |  | 2.04 |  | 13.09 |  | 36 |
| Difenoconazole | 38.68 | 18.33 | 6.64 | 138.89 | 119.59 | 3168.56 |
| Dimethomorph | 45.3 | 40.5 | 8.12 | 768.46 | 222.69 | 67656.1 |
| Dimoxystrobin | 10.9 | 0.9 | 0.48 | 1.9 | 2.26 | 3.72 |
| Epoxiconazole* | 28.42 | 9.05 | 11 | 99.81 | 101.95 | 1992.22 |
| Fenpropimorph* | 8.97 | 0.23 | 0.47 | 0.72 | 1.18 | 0.72 |
| Fluazinam | 0.43 | 1.36 |  | 2.38 |  | 6.12 |
| Fludioxonil | 2.56 | 3.85 | 15.64 | 1349.55 | 67.69 | 11801.67 |
| Fluopicolid | 28.85 | 23.76 | 15.72 | 63.15 | 219.54 | 2383.26 |
| Fluopyram | 93.59 | 70.36 | 6.04 | 28.79 | 164.62 | 954.42 |
| Iprovalicarb | 0.85 | 7.47 | 0.09 | 2.74 | 0.12 | 7.01 |
| Kresoxim methyl | 0.43 | 8.37 | 0.66 | 43.76 | 0.66 | 754.98 |
| Mandipropamid | 13.46 | 25.79 | 1.88 | 22.81 | 45.2 | 1451.95 |
| Metalaxyl | 21.79 | 28.28 | 0.34 | 4.04 | 5.8 | 100.65 |
| Metconazole | 3.42 | 1.13 | 2.9 | 46.28 | 17.03 | 211.67 |
| Metrafenone | 41.03 | 32.35 | 26.53 | 543.07 | 226.58 | 9950.09 |
| Myclobutanil | 24.36 | 15.38 | 5.44 | 45.99 | 108.75 | 825.76 |
| Paclobutrazol |  |  |  |  |  |  |
| Penconazole | 18.16 | 4.52 | 1.37 | 2.53 | 9.12 | 6.54 |
| Pencycuron | 1.07 |  | 0.29 |  | 0.33 |  |
| Picoxystrobin* |  |  |  |  |  |  |
| Prochloraz |  |  |  |  |  |  |
| Propamocarb | 3.21 | 2.04 | 0.24 | 0.68 | 0.49 | 1.97 |
| Proquinazid | 20.3 | 37.1 | 4.73 | 123.46 | 115.21 | 9570.27 |
| Pyraclostrobin | 42.31 | 7.92 | 0.25 | 2.82 | 1.14 | 66.4 |
| Pyrimethanil | 1.28 | 1.13 | 0.98 | 5.2 | 1.61 | 9.28 |
| Spiroxamine | 44.87 | 46.83 | 1.42 | 33.34 | 42.83 | 1022.96 |
| Tebuconazole | 20.94 | 3.85 | 2.83 | 123.3 | 20.32 | 781.49 |
| Trifloxystrobin |  | 5.88 |  | 0.24 |  | 0.77 |

Table S1 Overview of number and frequency of detected CUPs, the mean concentrations and the maximal detected concentrations in 468 soil and 442 vegetation samples. Empty cells mean no detections. Top 3 values per matrix, pesticide group and rubric (detection frequency, total mean concentration and maximum concentrations) are coloured in red. Substances which were not approved in 2021 are marked with an asterisk (*).

| **Herbicides** |  |  |  |  |  |  |
| --- | --- | --- | --- | --- | --- | --- |
| 2,4-D |  |  |  |  |  |  |
| Aminopyralid |  |  |  |  |  |  |
| Bentazone* | 0.43 |  |  |  |  |  |
| Bromoxynil |  |  |  |  |  |  |
| Carfentrazone-ethyl |  |  |  |  |  |  |
| Chloridazon* | 25.21 | 0.68 | 0.44 | 2.17 | 1.98 | 3.94 |
| Chlortoluron | 2.56 | 0.23 | 0.24 | 0.48 | 0.37 | 0.48 |
| Clomazone | 12.82 | 6.33 | 2.6 | 24.32 | 23.64 | 406.36 |
| Diflufenican | 11.32 | 2.94 | 0.92 | 1.18 | 4.3 | 2.69 |
| Dimethenamid-P | 21.37 | 2.49 | 0.53 | 7.33 | 8.27 | 34.54 |
| Ethofumesate | 5.98 | 1.36 | 9.09 | 18.81 | 162.14 | 64.07 |
| Flazasulfuron | 0.21 | 0.45 | 0.64 | 10.18 | 0.64 | 17.5 |
| Florasulam |  |  |  |  |  |  |
| Flufenacet | 6.41 | 14.71 | 0.24 | 1.34 | 0.68 | 4.18 |
| Fluroxypyr | 0.21 |  |  |  |  |  |
| Flurtamone* | 0.64 |  | 0.08 |  | 0.12 |  |
| Isoproturon* | 7.26 | 0.23 | 0.16 |  | 0.31 |  |
| Lenacil |  |  |  |  |  |  |
| MCPA |  |  |  |  |  |  |
| Mecoprop |  |  |  |  |  |  |
| Metamitron | 8.76 | 2.71 | 22.66 | 2.26 | 478.73 | 8.59 |
| Metazachlor | 21.37 | 2.04 | 0.38 | 2.32 | 4.75 | 8.14 |
| Metobromuron | 5.34 | 4.07 | 0.17 | 1.23 | 0.2 | 3.42 |
| Metolachlor-S | 19.44 | 1.81 | 14.07 | 2.37 | 521.25 | 8.65 |
| Metsulfuron-methyl |  |  |  |  |  |  |
| Napropamide | 5.77 | 0.23 | 0.41 | 0.24 | 0.77 | 0.24 |
| Pendimethalin | 28.21 | 16.06 | 24.22 | 463.34 | 981.18 | 23161.44 |
| Picloram |  |  |  |  |  |  |
| Propaquizafop |  | 0.9 |  | 4.17 |  | 7.81 |
| Propyzamide | 14.53 | 24.21 | 0.24 | 5.23 | 0.65 | 39.63 |
| Prosulfocarb | 14.1 | 35.07 | 0.27 | 4.05 | 0.78 | 28.15 |
| Quinmerac | 1.5 |  | 1.1 |  | 4.69 |  |
| Quizalofop* |  |  |  |  |  |  |
| Terbuthylazine | 28.21 | 12.22 | 2.34 | 11.8 | 205.1 | 218.52 |
| Tribenuron-methyl |  |  |  |  |  |  |
| Tritosulfuron | 0.21 | 0.23 | 20.57 | 9.15 | 20.57 | 9.15 |
| **Insecticides** |  |  |  |  |  |  |
| Acetamiprid | 1.71 | 0.9 | 0.13 | 3.97 | 0.22 | 6.74 |
| Chlorantraniliprole | 14.32 | 10.18 | 3.15 | 102.73 | 27.09 | 3988.48 |
| Clothianidin* | 10.9 | 1.58 | 1.3 | 44.21 | 3.96 | 203.47 |
| Cyantraniliprole | 6.84 | 2.71 | 10.34 | 41.87 | 93.86 | 364.98 |
| Dimethoate* |  |  |  |  |  |  |
| Etofenprox | 0.21 |  | 0.53 |  | 0.53 |  |
| Fenoxycarb* |  |  |  |  |  |  |
| Fenpyroximate | 0.64 |  |  |  |  |  |
| Flonicamid |  |  |  |  |  |  |
| Flupyradifurone |  |  |  |  |  |  |
| Hexythiazox |  |  |  |  |  |  |
| Imidacloprid* | 2.99 |  | 0.61 |  | 0.66 |  |
| Indoxacarb | 4.06 | 0.9 | 1.2 | 4.85 | 1.69 | 10.74 |
| Methiocarb* |  |  |  |  |  |  |
| Pirimicarb | 13.68 | 0.9 | 0.14 | 0.16 | 1.67 | 0.19 |
| Pymetrozine* | 1.5 |  | 0.49 |  | 0.81 |  |
| Spinosad | 8.55 | 0.9 | 0.36 | 10.39 | 2.07 | 14.57 |
| Sulfoxaflor |  | 0.23 |  | 1.53 |  | 1.53 |
| Tebufenozide | 0.21 | 0.23 |  | 1.5 |  | 1.5 |
| Thiacloprid* |  | 0.23 |  | 0.09 |  | 0.09 |
| Thiamethoxam* | 5.98 | 1.36 | 0.68 | 9.2 | 3.38 | 46.79 |

Table S2 Number (#) of CUP residues in topsoils and vegetation samples from the single sample sites per month (February 2021 until February 2022) and distance (in-field: -20, off-field: 1 (1 m from the field into the meadow), 5 (5 m), 20 (20 m)). "na" indicates that no sample was available.

***See separate excel-file.***

Table S3 Minima and Maxima in topsoil and vegetation samples for each distance and cultivation type based on linear models. For the peak detection, an algorithm was used, which identified local peaks based on the current value and the threshold delta (0.3). Not for all distances a minimum was observed (“n.a.”).

|  | **Vegetation** |  |  |  |  | **Topsoil** | | | |
| --- | --- | --- | --- | --- | --- | --- | --- | --- | --- |
|  | **Minima** | **CUP Number** | **Maxima** | **CUP Number** |  | **Minima** | **CUP Number** | **Maxima** | **CUP Number** |
|  | Month |  | Month |  |  | Month |  | Month |  |
| Arable sites | | | | | Arable sites | | | | |
| -20 | December | 3.68 | July | 11.46 | -20 | n.a. |  | October | 20.75 |
| 1 | December | 2.07 | July | 8.74 | 1 | June | 12.75 | February ‘21, | 17.06, |
|  |  |  |  |  |  |  |  | October | 13.22 |
| 5 | December | 1.76 | July | 7.63 | 5 | April, | 2.81, | March, | 4.20, |
|  |  |  |  |  |  | August, | 3.65, | June, | 4.72, |
|  |  |  |  |  |  | October, | 2.17, | September, | 5, |
|  |  |  |  |  |  | December | 1.67 | November | 2.3 |
| 20 | December | 1.78 | July | 7.55 | 20 | December | 1.26 | September | 2.09 |
| Vegetable sites | | | | | Vegetable sites | | | | |
| -20 | September, | 4.46 | July | 21.77 | -20 | May | 19.04 | February ‘21, | 21.11, |
|  | December | 4.997579 | October | 8.65 |  |  |  | October | 20.75 |
| 1 | October | 5.78 | July | 12.01 | 1 | n.a. |  | September | 13.69 |
| 5 |  |  | December | 6.27 |  | April | 7.45 | February ‘21, | 8.56, |
|  | n.a. |  | July | 9.28 | 5 |  |  | October | 9.32 |
| 20 | n.a. |  | July | 6.45 | 20 | May | 3.85 | February ‘21, | 7.04, |
|  |  |  |  |  |  |  |  | October | 5.01 |
| Viticulture sites | | | | | Viticulture sites | | | | |
| -20 | n.a. |  | August | 17.03 | -20 | May | 13.95 | February ‘21, | 15.54, |
|  |  |  |  |  |  |  |  | October | 16.9 |
| 1 | n.a. |  | July | 15.8 | 1 | May | 10.76 | February ‘21, | 12.77, |
|  |  |  |  |  |  |  |  | August | 12.1 |
| 5 | n.a. |  | August | 13.97 | 5 | May | 10.38 | February ‘21, | 11.98, |
|  |  |  |  |  |  |  |  | September | 13.28 |
| 20 | n.a. |  | August | 12.69 | 20 | May, | 3.26, | February ‘21, | 6.91, |
|  |  |  |  |  |  | September | 4.67 | August, | 7, |
|  |  |  |  |  |  |  |  | October | 6.41 |

Table S4 KL-results for the land-use types and months.

| **Pair** |  | **Mean KL-Divergence** | |
| --- | --- | --- | --- |
| **Opponent1** | **Oppponent2** | **Soil** | **Vegetation** |
| Arable Sites | Vegetables Sites | 0 | 0.15 |
| Arable Sites | Viticulture Sites | 0.01 | 0.23 |
| Vegetables Sites | Viticulture Sites | 0.01 | 0.06 |

Table S5 Overview of KL-results for temporal distribution of number of CUPs. KL-Divergence is shown for soil and vegetation between different pairs, within one crop type the differences between the distances are shown and the differences of the mean of the distances for the crop types are displayed.

| **Pair** | | **KL-Divergence** | |
| --- | --- | --- | --- |
| **Opponent1** | **Opponent2** | **Soil** | **Vegetation** |
| Arable Sites in-field | Arable Sites 1 m | 0.006 | 0.022 |
| Arable Sites in-field | Arable Sites 5 m | 0.058 | 0.129 |
| Arable Sites in-field | Arable Sites 20 m | 0.023 | 0.113 |
| Arable Sites 1 m | Arable Sites 5 m | 0.051 | 0.049 |
| Arable Sites 1 m | Arable Sites 20 m | 0.013 | 0.04 |
| Arable Sites 5 m | Arable Sites 20 m | 0.024 | 0.003 |
| Vegetable Sites in-field | Vegetable Sites 1 m | 0.013 | 0.062 |
| Vegetable Sites in-field | Vegetable Sites 5 m | 0.013 | 0.16 |
| Vegetable Sites in-field | Vegetable Sites 20 m | 0.011 | 0.142 |
| Vegetable Sites 1 m | Vegetable Sites 5 m | 0.003 | 0.027 |
| Vegetable Sites 1 m | Vegetable Sites 20 m | 0.013 | 0.019 |
| Vegetable Sites 5 m | Vegetable Sites 20 m | 0.011 | 0.002 |
| Viticulture Sites in-field | Viticulture Sites 1 m | 0.002 | 0.011 |
| Viticulture Sites in-field | Viticulture Sites 5 m | 0.001 | 0.017 |
| Viticulture Sites in-field | Viticulture Sites 20 m | 0.018 | 0.018 |
| Viticulture Sites 1 m | Viticulture Sites 5 m | 0.003 | 0.013 |
| Viticulture Sites 1 m | Viticulture Sites 20 m | 0.021 | 0.008 |
| Viticulture Sites 5 m | Viticulture Sites 20 m | 0.018 | 0.016 |
| Arable Sites in-field | Vegetable Sites in-field | 0.001 | 0.05 |
| Arable Sites in-field | Viticulture Sites in-field | 0.005 | 0.175 |
| Vegetable Sites in-field | Viticulture Sites in-field | 0.002 | 0.296 |
| Arable Sites 1 m | Vegetable Sites 1 m | 0.008 | 0.075 |
| Arable Sites 1 m | Viticulture Sites 1 m | 0.012 | 0.164 |
| Vegetable Sites 1 m | Viticulture Sites 1 m | 0.019 | 0.069 |
| Arable Sites 5 m | Vegetable Sites 5 m | 0.056 | 0.256 |
| Arable Sites 5 m | Viticulture Sites 5 m | 0.073 | 0.266 |
| Vegetable Sites 5 m | Viticulture Sites 5 m | 0.009 | 0.009 |
| Arable Sites 20 m | Vegetable Sites 20 m | 0.024 | 0.245 |
| Arable Sites 20 m | Viticulture Sites 20 m | 0.062 | 0.192 |
| Vegetable Sites 20 m | Viticulture Sites 20 m | 0.017 | 0.062 |

Table S6 Table with Mean and Standard Error of numbers of CUPs, averaged over the months for each culture and distance.

| **Matrix** | **Distance** | **Crop type** | **min** | **max** | **mean** | **sd** | **n** |
| --- | --- | --- | --- | --- | --- | --- | --- |
| soil | In-field | Arable Sites | 12 | 28 | 19.1 | 4.6 | 39 |
| soil | In-field | Vegetables Sites | 12 | 25 | 19.3 | 3.2 | 39 |
| soil | In-field | Viticulture Sites | 11 | 21 | 15.2 | 2 | 39 |
| soil | 1 | Arable Sites | 2 | 27 | 12.8 | 7.6 | 39 |
| soil | 1 | Vegetables Sites | 5 | 21 | 11.6 | 4.4 | 39 |
| soil | 1 | Viticulture Sites | 9 | 15 | 11.5 | 1.6 | 39 |
| soil | 5 | Arable Sites | 1 | 10 | 3.3 | 2.1 | 39 |
| soil | 5 | Vegetables Sites | 3 | 16 | 7.6 | 3.5 | 39 |
| soil | 5 | Viticulture Sites | 6 | 17 | 11.5 | 2.8 | 39 |
| soil | 20 | Arable Sites | 1 | 5 | 1.9 | 1.1 | 39 |
| soil | 20 | Vegetables Sites | 1 | 15 | 4.5 | 3.6 | 39 |
| soil | 20 | Viticulture Sites | 2 | 8 | 5.2 | 1.4 | 39 |
| vegetation | In-field | Arable Sites | 1 | 16 | 7.1 | 4.3 | 22 |
| vegetation | In-field | Vegetables Sites | 3 | 25 | 10.8 | 6.8 | 30 |
| vegetation | In-field | Viticulture Sites | 2 | 20 | 12.2 | 5.3 | 39 |
| vegetation | 1 | Arable Sites | 1 | 15 | 4.1 | 3.8 | 39 |
| vegetation | 1 | Vegetables Sites | 1 | 15 | 7.4 | 4 | 39 |
| vegetation | 1 | Viticulture Sites | 0 | 19 | 10 | 5.1 | 39 |
| vegetation | 5 | Arable Sites | 0 | 15 | 3.3 | 3.6 | 39 |
| vegetation | 5 | Vegetables Sites | 1 | 17 | 7.1 | 4.2 | 39 |
| vegetation | 5 | Viticulture Sites | 1 | 18 | 9.9 | 4.6 | 39 |
| vegetation | 20 | Arable Sites | 0 | 15 | 3.2 | 3.6 | 39 |
| vegetation | 20 | Vegetables Sites | 0 | 11 | 5 | 2.6 | 39 |
| vegetation | 20 | Viticulture Sites | 1 | 18 | 7.7 | 4.6 | 39 |

Table S7 Coefficients for the exponential functions (a*exp(b*x) +c) calculated for the course of CUPs with the distances for each cultivation type and in both matrices.

| **Cultivation type** | **a (y-scaling)** | **b (decrease)** | **c (background)** |
| --- | --- | --- | --- |
| ***Vegetation*** |  |  |  |
| Arable Sites | 3.51 | -1.45 | 3.23 |
| Vegetable Sites | 4.49 | -0.22 | 5 |
| Viticulture Sites | 3.82 | -0.15 | 7.67 |
| ***Soil*** |  |  |  |
| Arable Sites | 17.35 | -0.47 | 1.87 |
| Vegetable Sites | 14.23 | -0.52 | 4.46 |
| Viticulture Sites | 9.04 | -0.11 | 5.15 |

Table S8 Frequency of CUP detections at the different distances for the three investigated crop types. For all soil samples per crop type n=39, for all off-field vegetation samples per crop type n=39 and for in-field vegetation samples: arable sites n= 22, vegetable sites n= 30, viticulture sites n= 39).

***See separate excel-file.***

Table S9 Overview of the MRQs calculated for collembola and earthworms based on the measured environmental concentrations (MECs) of 93 CUPs in topsoils in cultivated areas (in-field) and off-field (1, 5 and 20 meters) of arable (n=3), vegetable (n=3) and viticulture sites (n=3). The mean and maximum MRQ for both soil organisms at each agricultural site and month are shown. No risk (white MRQ > 0.01, low risk (light grey) 0.01 < MRQ < 0.1, medium risk (grey) 0.1 < MRQ < 1, very high risk (black) MRQ > 1).

|  | **Collembola** | | | | | | **Earthworms** | | | | | |
| --- | --- | --- | --- | --- | --- | --- | --- | --- | --- | --- | --- | --- |
|  | Arable site | | Vegetable site | | Viticulture site | | Arable site | | Vegetable site | | Viticulture site | |
| Month | Mean RQT | Max RQT | Mean RQT | Max RQT | Mean RQT | Max RQT | Mean RQT | Max RQT | Mean RQT | Max RQT | Mean RQT | Max RQT |
|  |  |  |  |  |  |  |  |  |  |  |  |  |
| In-field (-20m) | | | | | | | | | | | | |
| Feb'21 | 0.34 | 0.51 | 0.18 | 0.43 | 0.04 | 0.05 | 0.28 | 0.43 | 0.24 | 0.31 | 0.36 | 0.39 |
| Mar'21 | 0.38 | 0.82 | 0.11 | 0.30 | 0.04 | 0.09 | 0.30 | 0.44 | 0.30 | 0.60 | 1.65 | 3.83 |
| Apr'21 | 0.53 | 0.83 | 0.41 | 1.17 | 0.13 | 0.25 | 0.40 | 0.73 | 0.53 | 0.70 | 0.61 | 1.44 |
| May'21 | 0.40 | 0.64 | 0.15 | 0.33 | 0.17 | 0.31 | 0.31 | 0.54 | 0.71 | 1.02 | 1.00 | 1.52 |
| Jun'21 | 0.70 | 1.02 | 0.32 | 0.85 | 1.60 | 2.30 | 0.92 | 1.82 | 1.10 | 2.03 | 1.39 | 2.03 |
| Jul'21 | 1.07 | 2.46 | 0.19 | 0.32 | 1.31 | 2.87 | 0.39 | 0.46 | 4.33 | 7.33 | 1.44 | 2.86 |
| Aug'21 | 0.49 | 1.08 | 0.16 | 0.47 | 2.80 | 6.61 | 0.26 | 0.32 | 0.24 | 0.62 | 2.04 | 5.02 |
| Sep'21 | 0.78 | 1.75 | 0.32 | 0.59 | 0.57 | 0.83 | 0.31 | 0.42 | 0.84 | 1.43 | 1.09 | 1.75 |
| Oct'21 | 0.77 | 1.49 | 1.22 | 3.20 | 0.63 | 1.12 | 0.36 | 0.45 | 2.14 | 4.23 | 1.04 | 1.31 |
| Nov'21 | 0.33 | 0.49 | 0.82 | 2.19 | 0.33 | 0.66 | 0.26 | 0.51 | 1.16 | 1.95 | 0.75 | 1.07 |
| Dec'21 | 0.42 | 0.66 | 0.35 | 0.90 | 0.53 | 0.80 | 0.20 | 0.35 | 0.83 | 1.18 | 1.17 | 1.94 |
| Jan'22 | 0.47 | 0.81 | 0.29 | 0.69 | 0.17 | 0.30 | 0.19 | 0.33 | 0.62 | 0.86 | 0.61 | 0.86 |
| Feb'22 | 0.18 | 0.30 | 0.35 | 0.86 | 0.09 | 0.21 | 0.11 | 0.17 | 0.43 | 1.00 | 0.48 | 0.69 |
| Off-field 1 m | | | | | | | | | | | | |
| Feb'21 | 0.15 | 0.33 | 0.07 | 0.21 | 0.01 | 0.02 | 0.15 | 0.36 | 0.04 | 0.07 | 0.10 | 0.17 |
| Mar'21 | 0.15 | 0.30 | 0.07 | 0.20 | 0.01 | 0.27 | 0.12 | 0.29 | 0.06 | 0.11 | 0.33 | 0.82 |
| Apr'21 | 0.14 | 0.27 | 0.05 | 0.15 | 0.16 | 0.22 | 0.12 | 0.30 | 0.05 | 0.10 | 0.24 | 0.26 |
| May'21 | 0.21 | 0.42 | 0.10 | 0.29 | 0.08 | 3.47 | 0.18 | 0.39 | 0.09 | 0.19 | 0.15 | 0.27 |
| Jun'21 | 0.20 | 0.50 | 0.07 | 0.22 | 1.36 | 1.26 | 0.17 | 0.48 | 0.08 | 0.17 | 0.64 | 1.49 |
| Jul'21 | 0.15 | 0.31 | 0.09 | 0.27 | 0.62 | 0.70 | 0.13 | 0.34 | 0.72 | 1.99 | 0.32 | 0.58 |
| Aug'21 | 0.11 | 0.24 | 0.07 | 0.16 | 0.48 | 0.74 | 0.11 | 0.29 | 1.78 | 4.18 | 0.26 | 0.48 |
| Sep'21 | 0.13 | 0.29 | 0.04 | 0.11 | 0.20 | 0.34 | 0.11 | 0.32 | 0.09 | 0.23 | 0.20 | 0.43 |
| Oct'21 | 0.14 | 0.30 | 0.07 | 0.20 | 0.12 | 0.41 | 0.11 | 0.28 | 0.09 | 0.23 | 0.19 | 0.32 |
| Nov'21 | 0.07 | 0.11 | 0.04 | 0.13 | 0.14 | 0.34 | 0.07 | 0.14 | 0.04 | 0.07 | 0.12 | 0.25 |
| Dec'21 | 0.10 | 0.23 | 0.03 | 0.09 | 0.17 | 0.16 | 0.10 | 0.28 | 0.03 | 0.05 | 0.13 | 0.28 |
| Jan'22 | 0.06 | 0.11 | 0.04 | 0.10 | 0.06 | 0.16 | 0.07 | 0.16 | 0.01 | 0.02 | 0.18 | 0.21 |
| Feb'22 | 0.06 | 0.10 | 0.01 | 0.02 | 0.08 | 0.07 | 0.05 | 0.13 | 0.01 | 0.01 | 0.11 | 0.18 |

*Table S9 (cont) Overview of the MRQs calculated for collembola and earthworms based on the measured environmental concentrations (MECs) of 93 CUPs in topsoils in cultivated areas (in-field) and off-field (1, 5 and 20 meters) of arable (n=3), vegetable (n=3) and viticulture sites (n=3). The mean and maximum MRQ for both soil organisms at each agricultural site and month are shown. No risk (white MRQ > 0.01, low risk (light grey) 0.01 < MRQ < 0.1, medium risk (grey) 0.1 < MRQ < 1, very high risk (black) MRQ > 1).*

| Off-field 5 m | | | | | | | | | | | | |
| --- | --- | --- | --- | --- | --- | --- | --- | --- | --- | --- | --- | --- |
| Feb'21 | 0.01 | 0.01 | 0.00 | 0.00 | 0.01 | 0.01 | 0.01 | 0.02 | 0.01 | 0.01 | 0.16 | 0.35 |
| Mar'21 | 0.00 | 0.01 | 0.00 | 0.01 | 0.00 | 0.01 | 0.01 | 0.01 | 0.01 | 0.01 | 0.09 | 0.14 |
| Apr'21 | 0.01 | 0.01 | 0.00 | 0.00 | 0.10 | 0.61 | 0.00 | 0.01 | 0.01 | 0.02 | 0.08 | 0.21 |
| May'21 | 0.00 | 0.01 | 0.00 | 0.00 | 0.16 | 0.48 | 0.00 | 0.01 | 0.01 | 0.01 | 0.06 | 0.10 |
| Jun'21 | 0.00 | 0.01 | 0.00 | 0.00 | 0.27 | 0.29 | 0.01 | 0.01 | 0.01 | 0.01 | 0.13 | 0.19 |
| Jul'21 | 0.02 | 0.05 | 0.00 | 0.00 | 0.15 | 0.25 | 0.02 | 0.07 | 0.01 | 0.02 | 0.08 | 0.11 |
| Aug'21 | 0.00 | 0.01 | 0.00 | 0.01 | 0.32 | 0.36 | 0.00 | 0.01 | 0.03 | 0.06 | 0.12 | 0.12 |
| Sep'21 | 0.01 | 0.03 | 0.00 | 0.00 | 0.12 | 0.17 | 0.01 | 0.03 | 0.01 | 0.01 | 0.06 | 0.09 |
| Oct'21 | 0.00 | 0.01 | 0.01 | 0.01 | 0.21 | 0.45 | 0.00 | 0.01 | 0.05 | 0.09 | 0.15 | 0.19 |
| Nov'21 | 0.00 | 0.01 | 0.00 | 0.01 | 0.09 | 0.09 | 0.00 | 0.00 | 0.02 | 0.03 | 0.05 | 0.05 |
| Dec'21 | 0.00 | 0.00 | 0.00 | 0.00 | 0.22 | 0.20 | 0.00 | 0.00 | 0.01 | 0.01 | 0.10 | 0.18 |
| Jan'22 | 0.00 | 0.01 | 0.00 | 0.00 | 0.06 | 0.16 | 0.00 | 0.01 | 0.00 | 0.01 | 0.08 | 0.11 |
| Feb'22 | 0.00 | 0.00 | 0.00 | 0.00 | 0.03 | 0.07 | 0.00 | 0.00 | 0.00 | 0.01 | 0.02 | 0.03 |
| Off-field 20 m | | | | | | | | | | | | |
| Feb'21 | 0.00 | 0.00 | 0.00 | 0.00 | 0.00 | 0.00 | 0.00 | 0.00 | 0.02 | 0.06 | 0.00 | 0.00 |
| Mar'21 | 0.00 | 0.00 | 0.00 | 0.00 | 0.00 | 0.00 | 0.00 | 0.00 | 0.00 | 0.01 | 0.01 | 0.01 |
| Apr'21 | 0.00 | 0.00 | 0.00 | 0.00 | 0.00 | 0.00 | 0.00 | 0.00 | 0.00 | 0.01 | 0.00 | 0.01 |
| May'21 | 0.00 | 0.00 | 0.00 | 0.00 | 0.00 | 0.00 | 0.00 | 0.00 | 0.00 | 0.01 | 0.00 | 0.00 |
| Jun'21 | 0.00 | 0.00 | 0.00 | 0.00 | 0.00 | 0.00 | 0.00 | 0.00 | 0.00 | 0.01 | 0.00 | 0.01 |
| Jul'21 | 0.00 | 0.00 | 0.00 | 0.00 | 0.00 | 0.00 | 0.00 | 0.00 | 0.00 | 0.01 | 0.00 | 0.01 |
| Aug'21 | 0.00 | 0.00 | 0.00 | 0.00 | 0.03 | 0.09 | 0.00 | 0.00 | 0.01 | 0.03 | 0.01 | 0.01 |
| Sep'21 | 0.00 | 0.00 | 0.00 | 0.00 | 0.00 | 0.00 | 0.00 | 0.00 | 0.02 | 0.06 | 0.00 | 0.00 |
| Oct'21 | 0.00 | 0.00 | 0.00 | 0.00 | 0.00 | 0.00 | 0.00 | 0.00 | 0.04 | 0.12 | 0.00 | 0.01 |
| Nov'21 | 0.00 | 0.00 | 0.00 | 0.00 | 0.00 | 0.00 | 0.00 | 0.00 | 0.00 | 0.01 | 0.00 | 0.00 |
| Dec'21 | 0.00 | 0.00 | 0.00 | 0.00 | 0.00 | 0.00 | 0.00 | 0.00 | 0.00 | 0.01 | 0.00 | 0.01 |
| Jan'22 | 0.00 | 0.00 | 0.00 | 0.00 | 0.00 | 0.00 | 0.00 | 0.00 | 0.00 | 0.01 | 0.00 | 0.00 |
| Feb'22 | 0.00 | 0.00 | 0.00 | 0.00 | 0.00 | 0.00 | 0.00 | 0.00 | 0.00 | 0.01 | 0.00 | 0.00 |

Table S10 MRQ and RQ max and declared percentage by RQ

|  | **Collembola** | | | |
| --- | --- | --- | --- | --- |
|  | Average MRQ  (n=39) | Average RQ,max (n=39) | Average of declared percentage by RQ, max (n=39) | contributing CUPs |
| Arable sites  (in-field) | 0.47 | 0.4 | 86 | epoxiconazole (F) |
| Vegetable sites  (in-field) | 0.32 | 0.23 | 69 | azoxystrobin (F), epoxiconazole (F), clomazone (H), pendimethalin (H), propyzamid (H), chlorantraniliprole (I), cyantraniliprole (I) |
| Viticulture (in-field) | 0.64 | 0.6 | 79 | cyflufenamid (F), fluopyram (F), fluopicolid (F), metrafenone (F), myclobutanil (F) |
|  | **Earthworms** | | | |
|  | Average MRQ (n=39) | Average RQ,max (n=39) | Average of declared percentage by RQ, max (n=39) | contributing CUPs |
| Arable sites  (in-field) | 0.33 | 0.16 | 46 | boscalid (F), difenoconazole (F), dimoxystrobin (F), epoxiconazole (F), ethofumesat (H), terbuthylazine (H) |
| Vegetable sites  (in-field) | 1.03 | 0.71 | 62 | azoxystrobin (F), boscalid (F), difenoconazole (F), clomazone (F), |
| Viticulture (in-field) | 1.03 | 0.67 | 62 | boscalid (F), difenoconazole (F), fluopyram (F) |
|  | **wild bees** | | | |
|  | Average MHQ  (n=39) | Average HQ,max (n=39) | Average of declared percentage by HQ, max (n=39) | contributing CUPs |
| Arable sites  (in-field) | 0.57 | 0.42 | 63 | boscalid (F), epoxiconazole (F), s-metolochlor (H), clothianidin (I), thiamtehoxam (I) |
| Vegetable sites  (in-field) | 1.66 | 1.59 | 69 | bosclaid (F), difenoconazole (F), epoxiconazole (F), pendimethalin (H), clothianidin (I), indoxacarb (I) |
| Viticulture (in-field) | 0.07 | 0.03 | 43 | fluopciolid (F), fluopyram (F), metrafenone (F), myclobutanil (F), penconazole (F) |

Table S11 Risk Quotient (RQ) values for collembola for all single topsoil samples. RQ was calulated by measured environmetnal concentraion / (no-observed effect concentration / assessment factopr of 10)). Na indicates missing toxicological endpoint values.

***See separate excel-file.***

Table S12 Risk Quotient (RQ) values for earthworms for all single topsoil samples. RQ was calulated by measured environmetnal concentraion / (no-observed effect concentration / assessment factopr of 10)). Na indicates missing toxicological endpoint values.

***See separate excel-file.***

Table S13 Mean hazard quotient (MHQ) (n=3) and the maximal MHQ based on the recorded concentrations of 93 CUPSs in in-field topsoil samples of arable, vegetable and viticulture site solitary wild bees with a surrogate LD50 (honey bee LD50)/10 [31], with LD50 data taken from PPDB) and an acute contact of 2.23 g of soil over 48 h. The numbers displayed with a black background are MHQs > 1, indicating a lethal hazard.

|  | Arable site | | Vegetable site | | Viticulture site | |
| --- | --- | --- | --- | --- | --- | --- |
|  | Mean  MHQ | Max  MHQ | Mean  MHQ | Max  MHQ | Mean  MHQ | Max  MHQ |
| Month |  |  |  |  |  |  |
|  | in-field | | | | | |
| Feb'21 | 0.37 | 0.49 | 0.70 | 2.08 | 0.05 | 0.05 |
| Mar'21 | 0.38 | 1.13 | 0.01 | 0.01 | 0.09 | 0.18 |
| Apr'21 | 0.71 | 1.80 | 0.35 | 0.52 | 0.04 | 0.07 |
| May'21 | 0.41 | 1.19 | 0.24 | 0.37 | 0.05 | 0.06 |
| Jun'21 | 1.75 | 3.90 | 0.08 | 0.12 | 0.08 | 0.12 |
| Jul'21 | 1.54 | 4.54 | 0.29 | 0.66 | 0.10 | 0.15 |
| Aug'21 | 0.33 | 0.94 | 0.64 | 1.91 | 0.14 | 0.29 |
| Sep'21 | 0.25 | 0.71 | 2.18 | 5.65 | 0.09 | 0.10 |
| Oct'21 | 0.49 | 0.74 | 7.87 | 22.69 | 0.08 | 0.09 |
| Nov'21 | 0.29 | 0.52 | 5.00 | 14.82 | 0.06 | 0.06 |
| Dec'21 | 0.63 | 1.28 | 1.32 | 3.93 | 0.08 | 0.12 |
| Jan'22 | 0.31 | 0.68 | 1.40 | 3.64 | 0.05 | 0.05 |
| Feb'22 | 0.01 | 0.01 | 1.55 | 4.09 | 0.05 | 0.05 |
|  | off-field 1 m | | | | | |
| Feb'21 | 0.11 | 0.33 | 0.04 | 0.12 | 0.02 | 0.03 |
| Mar'21 | 0.01 | 0.01 | 0.19 | 0.57 | 0.02 | 0.03 |
| Apr'21 | 0.01 | 0.01 | 0.01 | 0.02 | 0.02 | 0.02 |
| May'21 | 0.01 | 0.02 | 0.05 | 0.13 | 0.01 | 0.02 |
| Jun'21 | 0.01 | 0.01 | 0.01 | 0.04 | 0.03 | 0.05 |
| Jul'21 | 0.00 | 0.01 | 0.01 | 0.03 | 0.02 | 0.03 |
| Aug'21 | 0.01 | 0.01 | 0.22 | 0.63 | 0.02 | 0.03 |
| Sep'21 | 0.01 | 0.01 | 0.00 | 0.01 | 0.01 | 0.02 |
| Oct'21 | 0.01 | 0.01 | 0.16 | 0.48 | 0.01 | 0.02 |
| Nov'21 | 0.00 | 0.01 | 0.01 | 0.02 | 0.01 | 0.02 |
| Dec'21 | 0.01 | 0.01 | 0.00 | 0.01 | 0.01 | 0.02 |
| Jan'22 | 0.00 | 0.01 | 0.01 | 0.02 | 0.01 | 0.01 |
| Feb'22 | 0.00 | 0.00 | 0.00 | 0.00 | 0.01 | 0.02 |
|  | off-field 5 m | | | | | |
| Feb'21 | 0.00 | 0.00 | 0.00 | 0.00 | 0.03 | 0.06 |
| Mar'21 | 0.00 | 0.00 | 0.00 | 0.00 | 0.01 | 0.01 |
| Apr'21 | 0.00 | 0.00 | 0.00 | 0.00 | 0.01 | 0.02 |
| May'21 | 0.00 | 0.00 | 0.00 | 0.00 | 0.01 | 0.01 |
| Jun'21 | 0.00 | 0.00 | 0.00 | 0.00 | 0.01 | 0.02 |
| Jul'21 | 0.00 | 0.00 | 0.00 | 0.00 | 0.01 | 0.02 |
| Aug'21 | 0.00 | 0.00 | 0.00 | 0.00 | 0.02 | 0.03 |
| Sep'21 | 0.00 | 0.00 | 0.00 | 0.00 | 0.01 | 0.01 |
| Oct'21 | 0.00 | 0.00 | 0.00 | 0.00 | 0.01 | 0.02 |
| Nov'21 | 0.00 | 0.00 | 0.00 | 0.00 | 0.01 | 0.02 |
| Dec'21 | 0.00 | 0.00 | 0.00 | 0.00 | 0.01 | 0.02 |
| Jan'22 | 0.00 | 0.00 | 0.00 | 0.00 | 0.01 | 0.01 |
| Feb'22 | 0.00 | 0.00 | 0.00 | 0.00 | 0.01 | 0.01 |

Table S13 (cont.) Mean hazard quotient (MHQ) (n=3) and the maximal MHQ based on the recorded concentrations of 93 CUPSs in in-field topsoil samples of arable, vegetable and viticulture site solitary wild bees with a surrogate LD50 (honey bee LD50)/10 [31], with LD50 data taken from PPDB) and an acute contact of 2.23 g of soil over 48 h. The numbers displayed with a brown background are MHQs > 1, indicating a lethal hazard.

|  | off-field 20 m | | | | | |
| --- | --- | --- | --- | --- | --- | --- |
| Feb'21 | 0.00 | 0.00 | 0.00 | 0.00 | 0.00 | 0.00 |
| Mar'21 | 0.00 | 0.00 | 0.00 | 0.00 | 0.00 | 0.00 |
| Apr'21 | 0.00 | 0.00 | 0.00 | 0.00 | 0.00 | 0.00 |
| May'21 | 0.00 | 0.00 | 0.00 | 0.00 | 0.00 | 0.00 |
| Jun'21 | 0.00 | 0.00 | 0.00 | 0.00 | 0.00 | 0.00 |
| Jul'21 | 0.00 | 0.00 | 0.00 | 0.00 | 0.00 | 0.00 |
| Aug'21 | 0.00 | 0.00 | 0.00 | 0.00 | 0.00 | 0.00 |
| Sep'21 | 0.00 | 0.00 | 0.00 | 0.00 | 0.00 | 0.00 |
| Oct'21 | 0.00 | 0.00 | 0.00 | 0.00 | 0.00 | 0.00 |
| Nov'21 | 0.00 | 0.00 | 0.00 | 0.00 | 0.00 | 0.00 |
| Dec'21 | 0.00 | 0.00 | 0.00 | 0.00 | 0.00 | 0.00 |
| Jan'22 | 0.00 | 0.00 | 0.00 | 0.00 | 0.00 | 0.00 |
| Feb'22 | 0.00 | 0.00 | 0.00 | 0.00 | 0.00 | 0.00 |

Table S14 Hazard Quotients (HQ) values for wild bees for all single topsoil samples. HQ was calculated by (matrix exposure (2.23 g) * measured environmental concentration [ng/g])/ (honey bee LD50/10). Na indicates missing toxicological endpoint values

***See separate excel-file.***

Table S15 List of sampling dates for all 13 sampling periods at the sampling sites (n=9).

| **Sample site** | **Date of sampling** | **“Month”** |
| --- | --- | --- |
| O1, O2, O3 | 02.03.2021 | Feb 21 |
| A1, A2, A3 | 03.03.2021 | Feb 21 |
| V0, V1, V2 | 04.03.2021 | Feb 21 |
| O1, O2, O3 | 29.03.2021 | Mrz 21 |
| V1, V2, V3 | 30.03.2021 | Mrz 21 |
| A1, A2, A3 | 31.03.2021 | Mrz 21 |
| O1, O2, O3, V1 | 27.04.2021 | Apr 21 |
| V2, V3, A1, A2, A3 | 28.04.2021 | Apr 21 |
| O1, O2, O3 | 25.05.2021 | Mai 21 |
| V1, V2, V3 | 26.05.2021 | Mai 21 |
| A1, A2, A3 | 27.05.2021 | Mai 21 |
| O1, O2, O3 | 22.06.2021 | Jun 21 |
| A1, A2, A3 | 23.06.2021 | Jun 21 |
| V1, V2, V3 | 24.06.2021 | Jun 21 |
| O1, O2, O3, V1 | 20.07.2021 | Jul 21 |
| V2, V3, A1, A2, A3 | 21.07.2021 | Jul 21 |
| O1, O2, O3, V1 | 18.08.2021 | Aug 21 |
| V2, V3, A1, A2, A3 | 19.08.2021 | Aug 21 |
| V1, V2, V3 | 15.09.2021 | Sep 21 |
| O1, O2, O3 | 16.09.2021 | Sep 21 |
| A1, A2, A3 | 17.09.2021 | Sep 21 |
| O1, O2, O3, V1 | 13.10.2021 | Okt 21 |
| V2, V3, A1, A2, A3 | 14.10.2021 | Okt 21 |
| O1, O2, O3, V1 | 09.11.2021 | Nov 21 |
| V2, V3, A1, A2, A3 | 10.11.2021 | Nov 21 |
| O1, O2, O3, V1 | 07.12.2021 | Dez 21 |
| V2, V3, A1, A2, A3 | 09.12.2021 | Dez 21 |
| O1, O2, O3, V1 | 12.01.22 | Jan 22 |
| V2, V3, A1, A2, A3 | 13.01.22 | Jan 22 |
| O1, O2, O3, V1 | 24.02.22 | Feb 22 |
| V2, V3, A1, A2, A3 | 25.02.22 | Feb 22 |

Table S16 Cultivation sequence and soil types of the nine investigated fields and the adjacent meadow (off-field).

| **Culture** | **Site** | **Association of municipalities** | **Cultivation sequence 02/21 – 02/22** | **In-field soil type** | **Off-field soil type** | **Topographic** | **Note** |
| --- | --- | --- | --- | --- | --- | --- | --- |
|  |  |  |  |  |  |  |  |
| ***Arable sites*** | | | | | | |  |
| Winter wheat | A1 | Bad Bergzabern | Winter wheat | slightly loamy sand | slightly loamy sand | flat |  |
| Maize | A2 | Bad Bergzabern | Maize | loess loam, loess | loess loam, loess | flat |  |
| Sugar beet | A3 | Bad Bergzabern | Sugar beet, winter wheat | loess loam, loess | loess loam, loess | flat |  |
| ***Vegetable sites*** | | | | | | |  |
| Salat | O1 | Lingenfeld | Salat, celery, green manure | loamy sand, loess loam, loess | loamy sand | flat |  |
| Potato | O2 | Lingenfeld | Potato, cabbage | slightly loamy sand | slightly loamy sand | flat |  |
| Fennel | O3 | Lingenfeld | Fennel, celery, radish | slightly loamy sand | Humic sand | flat |  |
| ***Viticulture sites*** | | | | | | |  |
| Wine | V1 | Landau-Land | wine | loess loam, loess | loess loam, loess | flat |  |
| Wine | V2 | Ilbesheim | wine | clay and sandy loam and humic loam sand | humic loam Sand | hillside situation |  |
| Wine | V3 | Ilbesheim | wine | Sandy loam | Sandy loam | hillside situation | 20 m sampling point in a nature conservation area |

Table S16b Table of distances between the sample sites in kilometres. A1, A2, A3 belonging to arable crops: O1, O2, O3 to vegetable crops and V1, V2, V3 to viticulture management sites.

| **Distance**  **[km]** | **A1** | **A2** | **A3** | **O1** | **O2** | **O3** | **V1** | **V2** | **V3** |
| --- | --- | --- | --- | --- | --- | --- | --- | --- | --- |
| **A1** |  |  |  |  |  |  |  |  |  |
| **A2** | **3,4** |  |  |  |  |  |  |  |  |
| **A3** | **3,0** | **1,7** |  |  |  |  |  |  |  |
| **O1** | **41,8** | **40,5** | **41,5** |  |  |  |  |  |  |
| **O2** | **40,1** | **38,8** | **39,8** | **3,2** |  |  |  |  |  |
| **O3** | **39,1** | **37,8** | **38,8** | **4,5** | **1,5** |  |  |  |  |
| **V1** | **22,7** | **17,2** | **17,7** | **28,9** | **22,4** | **20,8** |  |  |  |
| **V2** | **19,7** | **17,5** | **18,3** | **31,0** | **23,8** | **22,8** | **2,1** |  |  |
| **V3** | **19,9** | **17,7** | **18,5** | **31,2** | **24,0** | **23,0** | **2,3** | **0,2** |  |

Table S17 Limit of detection (LOD) and limit of quantification (LOQ) for all 93 analytes.

|  | **Soil** | | **Vegetation** | |
| --- | --- | --- | --- | --- |
|  | LOD (µg/kg) | LOQ (µg/kg) | LOD (µg/kg) | LOQ (µg/kg) |
| *Fungicides* | | | | |
| Azoxystrobin | 0.006 | 0.018 | 0.01 | 0.02 |
| Benalaxyl | 0.004 | 0.013 | 0.01 | 0.04 |
| Bixafen | 0.03 | 0.1 | 0.02 | 0.06 |
| Boscalid | 0.1 | 0.3 | 0.14 | 0.43 |
| Cyazofamid | 0.05 | 0.14 | 0.06 | 0.19 |
| Cyflufenamid | 0.04 | 0.12 | 0.03 | 0.09 |
| Cymoxanil | 0.1 | 0.3 | 0.17 | 0.51 |
| Cyprodinil | 0.17 | 0.51 | 0.14 | 0.42 |
| Difenconazole | 0.1 | 0.4 | 0.14 | 0.44 |
| Dimethomorph | 0.05 | 0.14 | 0.04 | 0.12 |
| Dimoxystrobin | 0.01 | 0.03 | 0.02 | 0.05 |
| Epoxiconazole | 0.05 | 0.15 | 0.04 | 0.13 |
| Fenpropimorph | 0.11 | 0.34 | 0.16 | 0.47 |
| Fluazinam | 0.1 | 0.2 | 0.1 | 0.29 |
| Fludioxonil | 0.3 | 0.9 | 0.21 | 0.64 |
| Fluopicolid | 0.01 | 0.04 | 0.02 | 0.05 |
| Fluopyram | 0.01 | 0.03 | 0.01 | 0.04 |
| Iprovalicarb | 0.01 | 0.03 | 0.04 | 0.13 |
| Kresoxim-methyl | 0.1 | 0.4 | 0.1 | 0.32 |
| Mandipropamid | 0.02 | 0.07 | 0.02 | 0.08 |
| Metalaxyl-M | 0.01 | 0.03 | 0.01 | 0.02 |
| Metconazole | 0.06 | 0.18 | 0.11 | 0.33 |
| Metrafenone | 0.05 | 0.15 | 0.05 | 0.15 |
| Myclobutanil | 0.04 | 0.11 | 0.04 | 0.12 |
| Paclobutrazol | 0.09 | 0.28 | 0.07 | 0.23 |
| Penconazole | 0.03 | 0.08 | 0.04 | 0.13 |
| Pencycuron | 0.05 | 0.16 | 0.02 | 0.05 |
| Picoxystrobin | 0.02 | 0.05 | 0.02 | 0.06 |
| Prochloraz | 0.07 | 0.2 | 0.1 | 0.29 |
| Propamocarb | 0.002 | 0.05 | 0.003 | 0.01 |
| Proquinazid | 0.03 | 0.1 | 0.02 | 0.05 |
| Pyraclostrobin | 0.03 | 0.09 | 0.01 | 0.04 |
| Pyrimethanil | 0.07 | 0.2 | 0.08 | 0.24 |
| Spiroxamine | 0.03 | 0.08 | 0.01 | 0.03 |
| Tebuconazole | 0.14 | 0.43 | 0.12 | 0.37 |
| Trifloxystrobin | 0.02 | 0.05 | 0.01 | 0.03 |
| *Herbicides* | | | | |
| 2,4-D | 6.37 | 19.11 | 6.8 | 20.5 |
| Aminopyralid | 0.1 | 0.3 | 0.14 | 0.42 |
| Bentazone | 0.1 | 0.3 | 0.12 | 0.35 |
| Bromoxynil | 0.4 | 1.2 | 0.37 | 1.13 |
| Carfentrazone-ethyl | 0.2 | 0.7 | 0.28 | 0.86 |
| Chloridazon | 0.02 | 0.06 | 0.06 | 0.19 |
| Chlorotoluron | 0.02 | 0.06 | 0.02 | 0.06 |
| Clomazone | 0.03 | 0.09 | 0.02 | 0.06 |
| Diflufenican | 0.01 | 0.03 | 0.02 | 0.06 |
| Dimethenamid-P | 0.01 | 0.03 | 0.02 | 0.05 |
| Ethofumesate | 0.1 | 0.3 | 0.15 | 0.44 |
| Flazasulfuron | 0.05 | 0.14 | 0.04 | 1.2 |
| Florasulam | 0.07 | 0.21 | 0.12 | 0.38 |
| Flufenacet | 0.01 | 0.05 | 0.01 | 0.03 |
| Fluroxypyr | 3.8 | 11.4 | 3.7 | 11.1 |
| Flurtamone | 0.01 | 0.03 | 0.01 | 0.03 |
| Foramsulfuron | 0.2 | 0.6 | 0.28 | 0.86 |
| Isoproturon | 0.02 | 0.06 | 0.02 | 0.07 |
| Lenacil | 0.1 | 0.4 | 0.19 | 0.57 |
| MCPA | 1.3 | 3.8 | 1.91 | 5.78 |

Table S17 (cont) Limit of detection (LOD) and limit of quantification (LOQ) for all 93 analytes.

| Metamitron | 0.06 | 0.2 | 0.1 | 0.3 |
| --- | --- | --- | --- | --- |
| Metazachlor | 0.01 | 0.03 | 0.005 | 0.01 |
| Metobromuron | 0.01 | 0.04 | 0.04 | 0.13 |
| Metolachlor-S | 0.02 | 0.05 | 0.01 | 0.04 |
| Metsulfuron-methyl | 0.3 | 0.8 | 0.16 | 0.5 |
| Napropamide | 0.01 | 0.03 | 0.01 | 0.04 |
| Pendimethalin | 0.12 | 0.36 | 0.003 | 0.01 |
| Picloram | 5.6 | 17 | 7.1 | 21.5 |
| Propaquizafop | 0.01 | 0.04 | 0.01 | 0.04 |
| Propyzamide | 0.03 | 0.06 | 0.04 | 0.11 |
| Prosulfocarb | 0.02 | 0.05 | 0.01 | 0.02 |
| Quinmerac | 0.03 | 0.09 | 0.03 | 0.09 |
| Quizalofop-P | 2.91 | 8.83 | 2.28 | 6.92 |
| Terbuthylazine | 0.02 | 0.06 | 0.01 | 0.02 |
| Tribenuron-methyl | 0.02 | 0.06 | 0.03 | 0.08 |
| Tritosulfuron | 0.5 | 1.6 | 0.4 | 1.2 |
| *Insecticides* | | | | |
| Acetamiprid | 0.005 | 0.014 | 0.01 | 0.03 |
| Chlorantraniliprole | 0.04 | 0.13 | 0.04 | 0.11 |
| Clothianidin | 0.16 | 0.48 | 0.14 | 0.44 |
| Cyantraniliprole | 0.1 | 0.3 | 0.09 | 0.28 |
| Dimethoate | 0.02 | 0.05 | 0.02 | 0.06 |
| Etofenprox | 0.02 | 0.05 | 0.01 | 0.04 |
| Fenoxycarb | 0.03 | 0.09 | 0.04 | 0.12 |
| Fenpyroximate | 0.01 | 0.02 | 0.01 | 0.04 |
| Flonicamid | 0.34 | 1.01 | 0.41 | 1.23 |
| Flupyradifuron | 0.07 | 0.2 | 0.08 | 0.25 |
| Hexythiazox | 0.01 | 0.02 | 0.01 | 0.04 |
| Imidacloprid | 0.15 | 0.81 | 0.27 | 0.83 |
| Indoxacarb | 0.2 | 0.5 | 0.14 | 0.43 |
| Methiocarb | 0.01 | 0.03 | 0.02 | 0.05 |
| Pirimicarb | 0.003 | 0.008 | 0.06 | 0.17 |
| Pymetrozine | 0.06 | 0.2 | 0.02 | 0.05 |
| Spinosad | 0.11 | 0.32 | 0.12 | 0.35 |
| Sulfoxaflor | 0.06 | 0.17 | 0.04 | 0.11 |
| Tebufenozide | 0.02 | 0.05 | 0.03 | 0.09 |
| Thiacloprid | 0.01 | 0.03 | 0.01 | 0.02 |
| Thiamethoxam | 0.01 | 0.03 | 0.02 | 0.06 |

Table S18 Overview of chronic no-observed effect concentrations (NOECs) for earthworms and collembola obtained from PPDB, European Commission (values marked with an asterisk “*”) or literature (values marked with a degree sign “°”). “n.a.” indicating no values available.

|  | Springtails (*Folsomia candida*) | | Earthworms (*Eisenia foetida*) | |
| --- | --- | --- | --- | --- |
|  | NOEC [mg/kg] | LC50 [mg/kg] | NOEC [mg/kg] | LC50 [mg/kg] |
| **Fungicides** |  |  |  |  |
| Azoxystrobin | 25[1]* |  | 3 |  |
| Benalaxyl | 31.25 |  | 36.4 |  |
| Bixafen | 7.74 |  | 100 |  |
| Boscalid | 1000 |  | 1.197 |  |
| Cyazofamid | 37.4 |  | 2 |  |
| Cyflufenamid | 0.0178 |  | 0.667 |  |
| Difenconazole | 500 |  | 0.2 |  |
| Dimethomorph | 250[2]* |  | 60 |  |
| Dimoxystrobin | 1000[3]* |  | 0.089 |  |
| Epoxiconazole | 0.495 |  | 3.24 |  |
| Fenpropimorph | n.a. | n.a. | 4.7 |  |
| Fluazinam | 0.615 |  | 0.175 |  |
| Fludioxonil | 14.4 |  | 20 |  |
| Fluopicolid | 31.25 |  | 62.5 |  |
| Fluopyram | 103.8 |  | 11.42 |  |
| Iprovalicarb | 1000[4]* |  | 3.37 |  |
| Kresoxim methyl | n.a. | n.a. | n.a. | 469 |
| Mandipropamid | 20 |  | 16 |  |
| Metalaxyl_M | 125 |  | 35.63 |  |
| Metconazole | 160 |  | 20 |  |
| Metrafenone | 37.5 |  | 50.89 |  |
| Myclobutanil | 10[5]* |  | 10.3 |  |
| Penconazole | 49.4 |  | n.a. | 331.5 |
| Pencycuron | 60 |  | 3.3 |  |
| Propamocarb | 677[6]* |  | n.a. | n.a. |
| Proquinazid | 308.6 |  | 25.45 |  |
| Pyraclostrobin | 23.9 |  | 23.1 |  |
| Pyrimethanil | 23.19 |  | 4.12 |  |
| Spiroxamine | 32 |  | 5 |  |
| Tebuconazole | 250 |  | 10 |  |
| **Herbicides** |  |  |  |  |
| Bentazone | n.a. | n.a. | n.a. | 1000 |
| Chloridazon | n.a. | n.a. | n.a. | 1000 |
| Chlortoluron | 195 |  | 31.25 |  |
| Clomazone | 11.3 |  | 0.8 |  |
| Diflufenican | 5000 |  | n.a. | 500 |
| Dimethenamid-P | 12.5 |  | 25.4 |  |
| Ethofumesate | 13.35 |  | 4.42 |  |
| Flazasulfuron | 125 |  | 8 |  |
| Flufenacet | 31.5 |  | 1.2 |  |
| Fluroxypyr | n.a. | n.a. | 3.05 |  |
| Flurtamone | 500 |  | 47.5 |  |
| Isoproturon | 24.3[7]* |  | 10000 |  |

Table S18 (cont) Overview of chronic no-observed effect concentrations (NOECs) for earthworms and collembola obtained from PPDB, European Commission (values marked in yellow) or literature (values marked in green). “n.a.” indicating no values available or searched for.

| Metamitron | 22.4 |  | 28 |  |
| --- | --- | --- | --- | --- |
| Metazachlor | 205 |  | 2.31 |  |
| Metobromuron | 23.66 |  | 233 |  |
| Metolachlor-S | n.a. | n.a. | 26.65 |  |
| Napropamide | n.a. | n.a. | 30 |  |
| Pendimethalin | 193 |  | 33.45 |  |
| Propyzamide | 1.4 |  | 3.3 |  |
| Prosulfocarb | n.a. | n.a. | n.a. | 71.8 |
| Quinmerac | 1000 |  | 0.775 |  |
| Terbuthylazine | n.a. | n.a. | n.a. | 141.7 |
| Tritosulfuron | n.a. | n.a. | 20 |  |
| **Insecticides** |  |  |  |  |
| Acetamiprid | 0.27 |  | 20 |  |
| Chlorantraniliprole | 0.39 |  | 20 |  |
| Clothianidin | 0.093[8]° |  | 2.5 |  |
| Cyantraniliprole | 0.415[8]° |  | n.a. | 945 |
| Etofenprox | n.a. | n.a. | n.a. | 24.6 |
| Fenpyroximate | 25 |  | 2.71 |  |
| Imidacloprid | 0.44[8]° |  | 0.178 |  |
| Indoxacarb | 125 |  | 29.2 |  |
| Pirimicarb | 33 |  | 5.46 |  |
| Pymetrozine | 1000 |  | 1.386 |  |
| Spinosad | 4.3[9]* |  | n.a. |  |
| Tebufenozide | 7.21[10]* | . | 1000 |  |
| Thiamethoxam | 0.34[8]° |  | 5.34 |  |

*Table S19 Overview of chronic no-observed effect concentrations (NOECs) for earthworms and collembola obtained from PPDB.*

| *Table Lethal Dosis 50 (LD50) values for Honey bees, values were obtained from PPDB.* | | | |
| --- | --- | --- | --- |
| CUP | Honey bee contact acute LD50 ng/bee | CUP | Honey bee contact acute LD50 ng/bee |
| *Fungicides* |  |  |  |
| Azoxystrobin | 200000 | Ethofumesate | 100000 |
| Benalaxyl | 100000 | Flazasulfuron | 100000 |
| Bixafen | 121400 | Florasulam | 109200 |
| Boscalid | 200000 | Flufenacet | 180000 |
| Cyazofamid | 100000 | Fluroxypyr | 100000 |
| Cyflufenamid | 100000 | Flurtamone | 100000 |
| Cymoxanil | 100000 | Isoproturon | 200000 |
| Cyprodinil | 75000 | Lenacil | 227200 |
| Difenconazole | 100000 | MCPA | 200000 |
| Dimethomorph | 102000 | Mecoprop | 100000 |
| Dimoxystrobin | 100000 | Metamitron | 100000 |
| Epoxiconazole | 100000 | Metazachlor | 100000 |
| Fenpropimorph | 100000 | Metobromuron | 200000 |
| Fluazinam | 200000 | Metolachlor-S | 110000 |
| Fludioxonil | 100000 | Metsulfuron-methyl | 50000 |
| Fluopicolid | 100000 | Napropamide | 100000 |
| Fluopyram | 100000 | Pendimethalin | 100000 |
| Iprovalicarb | 200000 | Picloram | 83500 |
| Kresoxim methyl | 100000 | Propaquizafop | 200000 |
| Mandipropamid | 200000 | Propyzamide | 136000 |
| Metalaxyl | 100000 | Prosulfocarb | 80000 |
| Metconazole | 100000 | Quinmerac | 100000 |
| Metrafenone | 100000 | Quizalofop | 100000 |
| Myclobutanil | 33900 | Terbuthylazine | 32000 |
| Paclobutrazol | 40000 | Tribenuron-methyl | 98400 |
| Penconazole | 3000 | Tritosulfuron | 200000 |
| Pencycuron | 100000 | *Insecticides* |  |
| Picoxystrobin | 200000 | Acetamiprid | 8090 |
| Prochloraz | 141300 | Chlorantraniliprole | 4000 |
| Propamocarb | 100000 | Clothianidin | 44 |
| Proquinazid | 197000 | Cyantraniliprole | 93.4 |
| Prothioconazole | 100000 | Dimethoate | 100 |
| Pyraclostrobin | 100000 | Etofenprox | 38 |
| Pyrimethanil | 4200 | Fenoxycarb | 204000 |
| Spiroxamine | 200000 | Fenpyroximate | 15800 |
| Tebuconazole | 200000 | Flonicamid | 100000 |
| Trifloxystrobin | 100000 | Flupyradifurone | 200000 |
| *Herbicides* |  | Hexythiazox | 200000 |
| 2,4-D | 6260 | Imidacloprid | 81 |
| Aminopyralid | 200000 | Indoxacarb | 80 |
| Bentazone | 150000 | Methiocarb | 230 |
| Bromoxynil | 81000 | Pirimicarb | 17800 |
| Carfentrazone-ethyl | 200000 | Pymetrozine | 100000 |
| Chloridazon | 200200 | Spinosad | n.a. |
| Chlortoluron | 89500 | Sulfoxaflor | 379 |
| Clomazone | 100000 | Tebufenozide | 234000 |
| Diflufenican | 200000 | Thiacloprid | 38820 |
| Dimethenamid-P | 50000 | Thiamethoxam | 24 |

**Figures**


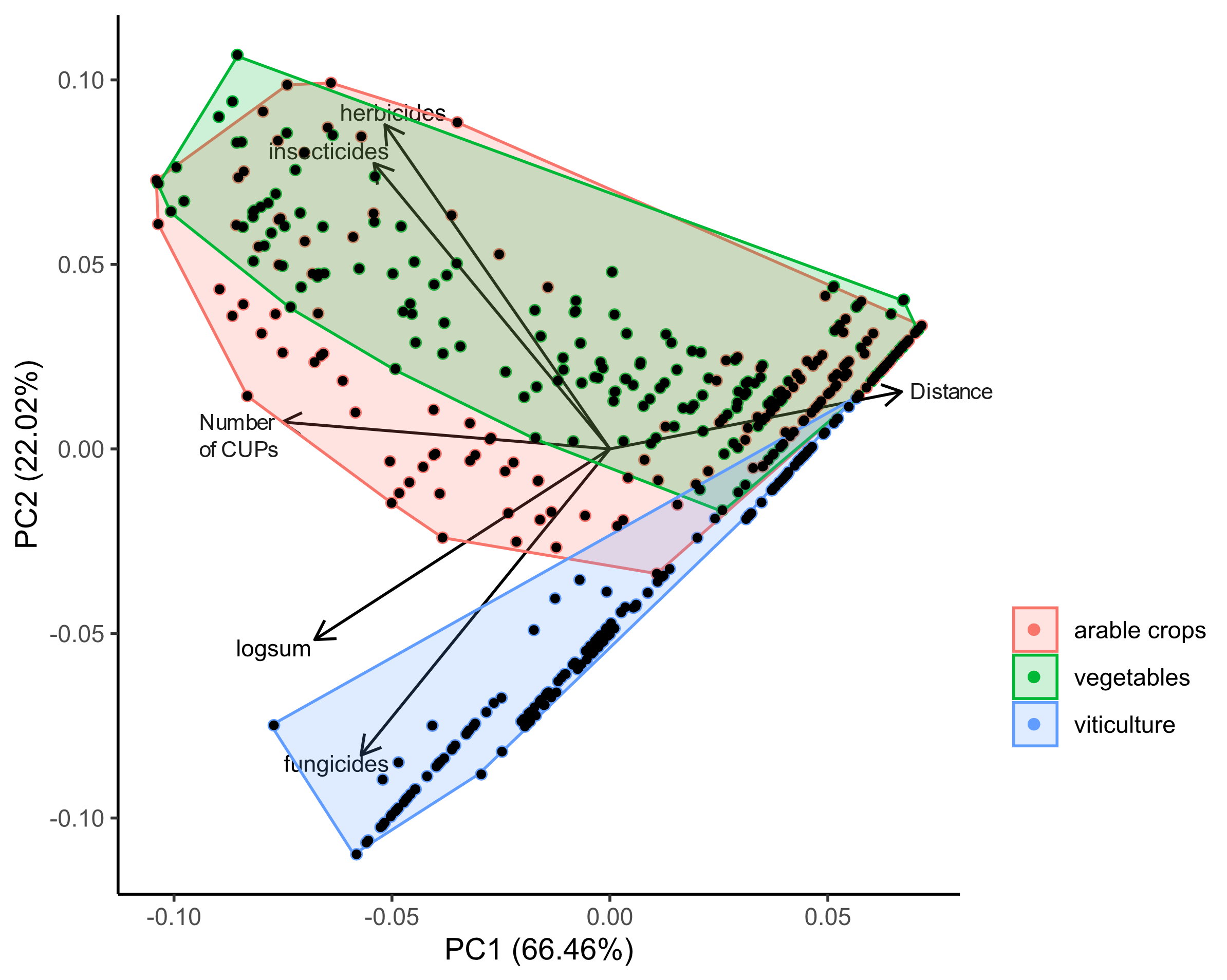


a


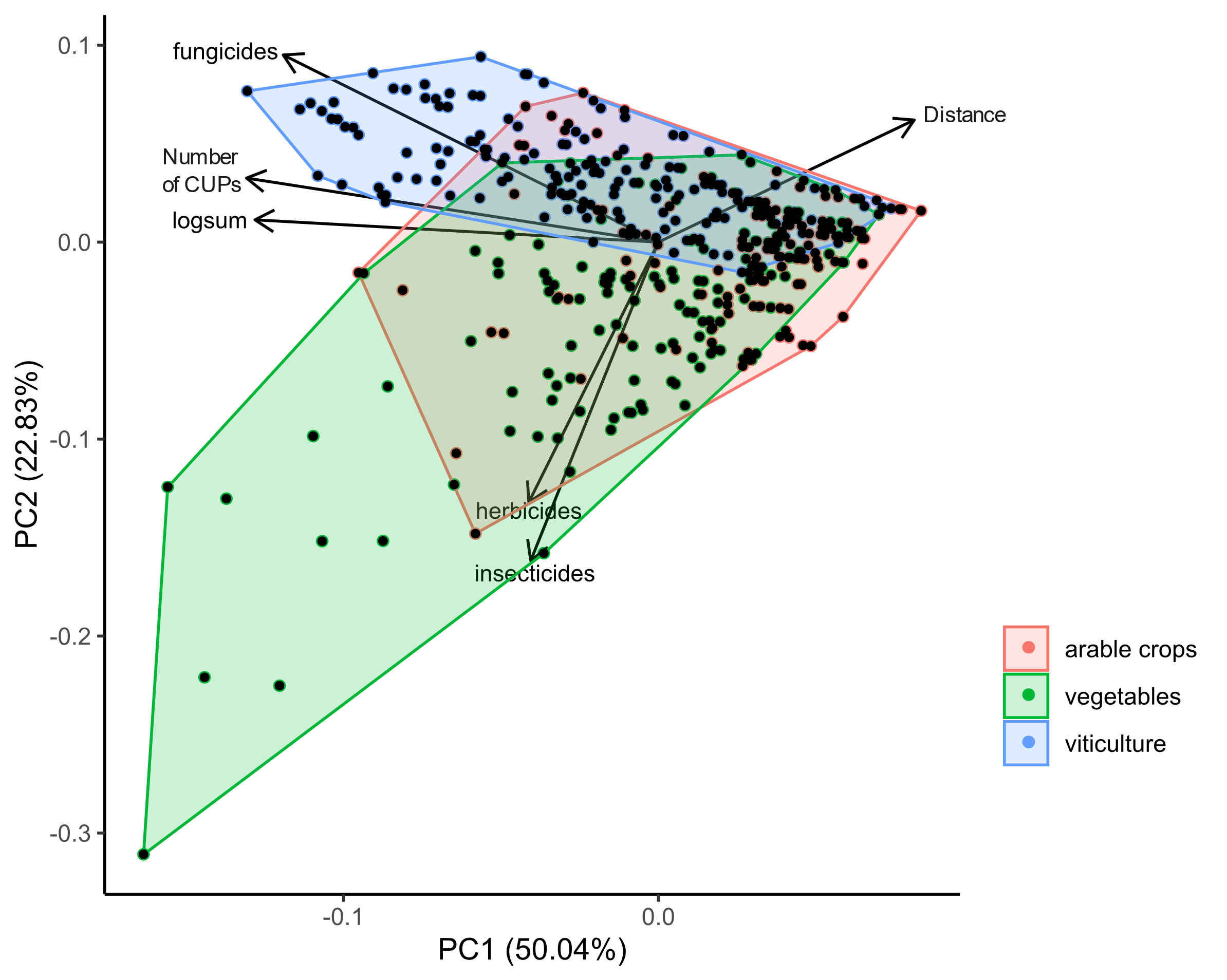


b

Figure S1 [a] Principal Component Analysis of Soil data. And [b] of Vegetation data. PCA biplots showing the relation between six variables for all data points represented by two principal components (PCs). As vectors: number of CUPs from herbicide groups (“herbicides”), number of CUPs from fungicides groups (“fungicides”), number of CUPs from insecticide groups (“insecticides”), total number of CUPs per sample (“Number of CUPs”), logarithmic cumulative concentration per sample (“logsum”), distance (field, 1, 5 and 20 m in a meadow, “Distance”). Cumulative explained variance: 88,48% for soil PCA and 72,87% for vegetation data. Land-use types were clustered.

**Methods**

**Sample processing & extraction**

After transport to the laboratory, soil samples were frozen at -20 °C before freeze drying for 48 h (Alpha 1-4 LSCbasic, Christ, Osterode, Germany). Freeze dried samples were sieved at 2 mm (Standard-compliant test sieve, ISO 3310-1, Haver & Boecker OHG, Oelde, Germany) and stored at -20°C until extraction. For extraction, 5 g ± 0.01 g of sieved soil was weighed (ME802, Mettler Toledo, Ohio, USA, d = 0.01 g) into 50 mL tubes. To compensate any interferences matrix-matched external calibration was used. Matrix-matched standards were prepared out of soil and vegetation samples (Jockgrim, Germany, 49°05'10" N, 8°15'33" E) known to not contain measurable pesticide concentrations (blank soil and vegetation samples). For quality control, all samples were spiked with 50 µL of deuterated standard imidacloprid-D4 (98.9 %, Dr. Ehrenstorfer GmbH, Augsburg, Germany) in acetonitrile (MeCN, HPLC gradient grade ≥ 99.9 %, Honeywell, Charlotte, USA) (concentration: 10 mg/L). The solvent was allowed to evaporate for 30 min under a fume hood. A respective blank sample was spiked with imidacloprid-D4 after extraction (concentration: 10 mg/L). The peak area of imidacloprid-D4 in samples and of the respective blank sample were compared, to check for extraction quality.

For the salt extraction of soil samples, 5 g of ammonium formate (NH4HCO2, reagent grade ≥ 99.0 %, Sigma-Aldrich, St. Louis, USA) and 10 mL of 2.5 % formic acid (CH2O2, HiPerSolv Chromanorm ≥ 99.0 %, VWR, Radnor, USA) in MeCN were added. The tubes were closed thoroughly and were shaken in an overhead shaker for 60 min (Stuart drive Rotator drive STR4, Cole-Parmer, Vernon Hills, USA). Afterwards, tubes were centrifuged (MegaStar 1.6R, VWR, Radnor, USA) for 6 min at 3000 rpm. The supernatant was further decanted through 2 μm filters (17 mm HPLC syringe filter, PTFE, hydrophobic, BGB Analytik, Lörrach, Germany) into 20 ml glass tubes. If not measured directly, extracts were stored at -20 °C until measurement. After freezing, analytical measurement samples were vortexed and filtered again at 2 μm into HPLC-vials.

Vegetation samples were air-dried at room temperature, frozen at -20 °C and then freeze-dried. Dried vegetation samples were grinded in a knife mill (GRINDOMIX GM 300, Retsch, Haan, Germany) for 2 x 1 min at 3000 rpm. Grinded vegetation was stored in small freezer bags at -20°C until extraction. For extraction, 1 g ± 0.01 g of grinded vegetation was weighed (ME802, Mettler Toledo, Ohio, USA, d = 0.01 g) into 50 mL tubes. Like soil samples, vegetation was spiked with Imidacloprid-D4 (25 µL Imidacloprid-D4 in MeCN, concentration: 10 mg/L) and the solvent was allowed to evaporate for 15 min. For the salt extraction, 0.25 g of NH4HCO2 and 10 mL of 2.5 % CH2O2 in MeCN were added. Subsequent overhead shaking, centrifuging and filtering followed the methodology as described for soil samples. Before analytical measurement, chlorophyll in vegetation extracts necessitates an additional purification step by dispersive solid phase extraction (dSPE) (Schenck et al., 2002). For this, 1 mL of sample extract was added to 7.5 mg graphitized carbon black (GCB, Carbon SPE Bulk Sorbent, Agilent Technologies, Santa Clara, USA) in 2 mL Eppendorf tubes. The tubes were vortexed vigorously for 60 s and the supernatant filtered through 2 μm filters directly into the measurement vials.

**CUP residue analysis**

The 96 CUPs were selected based on use records of the Julius Kühn-Institute (JKI), Germany. CUPS were selected that were frequently applied in winter wheat, oilseed rape, maize, potatoes and wine in 2016 and 2017. Additionally, newer insecticides such as chlorantraniliprole were included together with pesticides that were regularly detected in German small streams. Glyphosate and its metabolite AMPA were not included in the analytical portfolio. For quality control samples, a standard mix solution containing 96 analytes (24 I, 36 F, 36 H) in concentrations of 5 mg L 1 was prepared. For stock solution preparation, single component analytical standards (Dr. Ehrenstorfer GmbH, Augsburg, Germany and Sigma Aldrich, St. Louis, USA) were separately dissolved in MeCN to 400 mg/L. The single substance stock solutions were combined with multi-component fungicide, herbicide and insecticide solutions purchased from Restek (Bellefonte, USA, concentration = 100 mg/L) as indicated in Table A4, resulting in the final mix solution containing all analytes in concentrations of 5 mg/L in MeCN. CUP standards and working solutions were stored at -20°C.

The quantification of CUP concentrations in all samples was performed using external matrix-matched calibrations. For calibration standards, blank soil or blank vegetation extracts, respectively, were fortified with the multi-analyte standard mix solution resulting in calibration concentrations of 0.025, 0.05, 0.1, 0.5, 1, 10, 50 and 100 μg/L. The measurement of standards was repeated every 20 samples in order to account for possible system deviations during the run time. The final calibration curve for quantification was calculated as mean of three calibration standard measurements (i.e., providing 24 calibration points at 8 concentration levels).

For the positive confirmation of an analyte following criteria were considered: The retention time of the peak had to match the standard sample in a similar concentrated calibration standard. The qualifier ion ratios had to lay in ranges of 70 – 130 %. Pesticides detected below the determined LOD, were classified as non-detected, all others as detected. For counting detected CUPs also residues <LOQ were considered, for concentration specific questions only measured residues (>LOQ) were used. A blank sample of MeCN, MeCN plus FA as well as of the respective matrix extract was included in each measurement run to check for possible contamination. A matrix matched signal suppression or enhancement was excluded for all relevant analytes by comparison of analytical response of spiked blank samples to standards in MeCN. HPLC-MS/MS data was processed with Agilent Mass Hunter Quantitative Analysis software (Version 10.0., Build 10.0.707.0, Agilent Technologies, 2006 – 2018, Santa Clara CA, USA). A weighting factor of 1/x2 was used to weight the calibration curve if necessary, shown to be best for bioanalytical data [11], to improve curve fit and quantification results for low concentrations [12].

**KL-Divergence**

Individual loess smooths were compared with the Kullback-Leibler (KL) divergence. In preparation, the smooth values were divided by their own sum to obtain a probability distribution with a sum of 1. The KL-divergence was calculated as

KL(P‖Q)=∑P(P)*log2(P(P)/P(Q)) 〗=H(P,Q)-H(P),

where H(P,Q) denotes the joint entropy of the probability distributions P and Q and H(P) denotes the entropy of probability distribution P [13]. Due to the directional dependence of the comparison between P and Q, the mean was calculated of both directions. This mean KL-value was used to compare the two distributions, whereby comparisons of distributions with smaller result values are more similar (less entropy is lost) than comparisons of distributions with larger result values. The value can vary between 0 and 1. This comparison looks at the progression or shape of the curves, but not the actual density of the y-values.

**Loess smoothing**

A Loess smooth was used to obtain a coherent progression over the year and distance to the field margin [14]. A smoothing parameter is required, which determines how sensitively the curve reacts to outliers, short-term fluctuations and background noise. The function loess.as (package:fancova) was used to automatically select this parameter using generalized cross validation [15]. A smoothing value between 0.05 and 0.95 was tested. Each Loess smooth was then performed with its selected smoothing parameter. Smooth values close to 0 have sometimes become negative, these have been set to 0.

**Generalised linear models**

Additionally, we analysed the relationship between the predictors management systems (arable crops, vegetable crops, viticulture), distance (-20, 1, 5, 20) and month (February 2021 to February 2022) and the number and sum concentration of CUPs using generalised linear models (GLMs). For models with the number of CUPs as response variable, we used a quasipoisson distribution to account for overdispersion, which was previously tested with dispersion test (AER package [16]). For the sum concentration we calculated the logarithm after adding a pseudo constant of 0.1 to approach a Gaussian distribution and homogeneity of variance was further assumed as a prerequisite for GLMs. All predictors were used as qualitative factors inside the GLM. We performed type II ANOVA tests to evaluate the significance of predictors. Each combination of management system, month and distance had a replication of 3 (three locations each).

Table S20 Relationships between the predictors management system (arable crops, vegetable crops, viticulture), distance (in-field, 1, 5, 20) and month (February 2021 to February 2022) and the response variables number and sum concentration of CUPs using generalized linear models (GLMs) for soil and vegetation. Statistic: the value of the test statistic used to assess the significance of a factor, df: degrees of freedom and the p-value.

| **predictors** | **statistic** | **df** | **p.value** | **response** | **dataset** |
| --- | --- | --- | --- | --- | --- |
| managment system | 13.2 | 2 | 1.35E-03 | num | Soil |
| distance | 732 | 3 | 2.21E-158 | num | Soil |
| month | 36.3 | 12 | 2.91E-04 | num | Soil |
| managment system | 336 | 2 | 9.61E-74 | logsum | Soil |
| distance | 1515 | 3 | 0 | logsum | Soil |
| month | 44.6 | 12 | 1.21E-05 | logsum | Soil |
| managment system | 247 | 2 | 2.72E-54 | num | Veg |
| distance | 90 | 3 | 2.23E-19 | num | Veg |
| month | 396 | 12 | 2.72E-77 | num | Veg |
| managment system | 193 | 2 | 1.02E-42 | logsum | Veg |
| distance | 208 | 3 | 9.57E-45 | logsum | Veg |
| month | 129 | 12 | 9.31E-22 | logsum | Veg |

References

1. European Commission. Azoxystrobin. ACB procedures - evaluation template **3** (2009).

2. European Commission. Dimethomorph Volume 1 (2017).

3. European Commission. Dimoxystrobin Volume 3 - B.9 (AS) (2017).

4. European Union. Iprovalicarb Volume 1 **1** (2013).

5. European Commission. Myclobutanil **1** (2006).

6. European Commission. Propamocarb **1** (2017).

7. European Commission. Isoproturon **3** (2014).

8. Martin, W. J., Sibley, P. K. & Prosser, R. S. Comparison of Established and Novel Insecticides on Survival and Reproduction of Folsomia candida. *Environmental toxicology and chemistry* **42,** 1516–1528 (2023).

9. European Commission. Spinosad (2017).

10. European Commission. Tebufenozide **3** (2006).

11. Almeida, A. M., Castel-Branco, M. M. & Falcão, A. C. PII: S1570-0232(02)00244-1 // Linear regression for calibration lines revisited: weighting schemes for bioanalytical methods. *Journal of chromatography. B, Analytical technologies in the biomedical and life sciences* **774,** 215–222 (2002).

12. Dolan, J. Calibration Curves, Part V: Curve Weighting **27,** 534–540 (2009).

13. Drost, H. Philentropy: Information Theory and Distance Quantification with R. *The Journal of Open Source Software* **3** (2018).

14. Jacoby, W. G. Loess: a nonparametric, graphical tool for depicting relationships between variables. *Electoral Studies* **19,** 577–613 (2000).

15. Wang, X. fANCOVA: Nonparametric Analysis of Covariance. R package version 0.6-1., 2020.

16. Kleiber, C. & Zeileis, A. *Applied Econometrics with R [R package AER version 1.2-14]* (Springer-Verlag, 2008).
